# Iron-catalyzed domino coupling reactions of π-systems

**DOI:** 10.3762/bjoc.17.196

**Published:** 2021-12-07

**Authors:** Austin Pounder, William Tam

**Affiliations:** 1 Guelph-Waterloo Centre for Graduate Work in Chemistry and Biochemistry, Department of Chemistry, University of Guelph, Guelph, Ontario, N1G 2W1, Canada

**Keywords:** cascade, catalysis, coupling, earth-abundant, iron

## Abstract

The development of environmentally benign, inexpensive, and earth-abundant metal catalysts is desirable from both an ecological and economic standpoint. Certainly, in the past couple decades, iron has become a key player in the development of sustainable coupling chemistry and has become an indispensable tool in organic synthesis. Over the last ten years, organic chemistry has witnessed substantial improvements in efficient synthesis because of domino reactions. These protocols are more atom-economic, produce less waste, and demand less time compared to a classical stepwise reaction. Although iron-catalyzed domino reactions require a mindset that differs from the more routine noble-metal, homogenous iron catalysis they bear the chance to enable coupling reactions that rival that of noble-metal-catalysis. This review provides an overview of iron-catalyzed domino coupling reactions of π-systems. The classifications and reactivity paradigms examined should assist readers and provide guidance for the design of novel domino reactions.

## Introduction

Over the past couple decades, the use of transition-metal-catalyzed cross-coupling reactions have become a staple within the organic chemist’s arsenal of carbon–carbon and carbon–heteroatom bond-forming reactions. Catalysis, as a synthetic tool, is widely employed to accomplish transformations to produce many various pharmaceuticals, polymeric materials, and fine chemicals [1–8]. Catalysis is one of the fundamental pillars of green chemistry, the design of chemical products and processes that reduce or eliminate the use and generation of hazardous substances, as well as increase the atom economy of the reaction [9]. Among the transition-metal (TM) catalysts often used, the late transition metals like rhodium [10–14], palladium [15–19], nickel [20–23], and iridium [24–27] have taken center stage when it comes to the development of synthetic methodology. Although these late TMs have contributed enormously to the various fields of organic, inorganic, and organometallic chemistry, growing concerns regarding their economic and ecological impacts have risen. This has prompted interest into the use of cheap, benign, and readily available first-row TMs [28–34].

**Figure 1:**
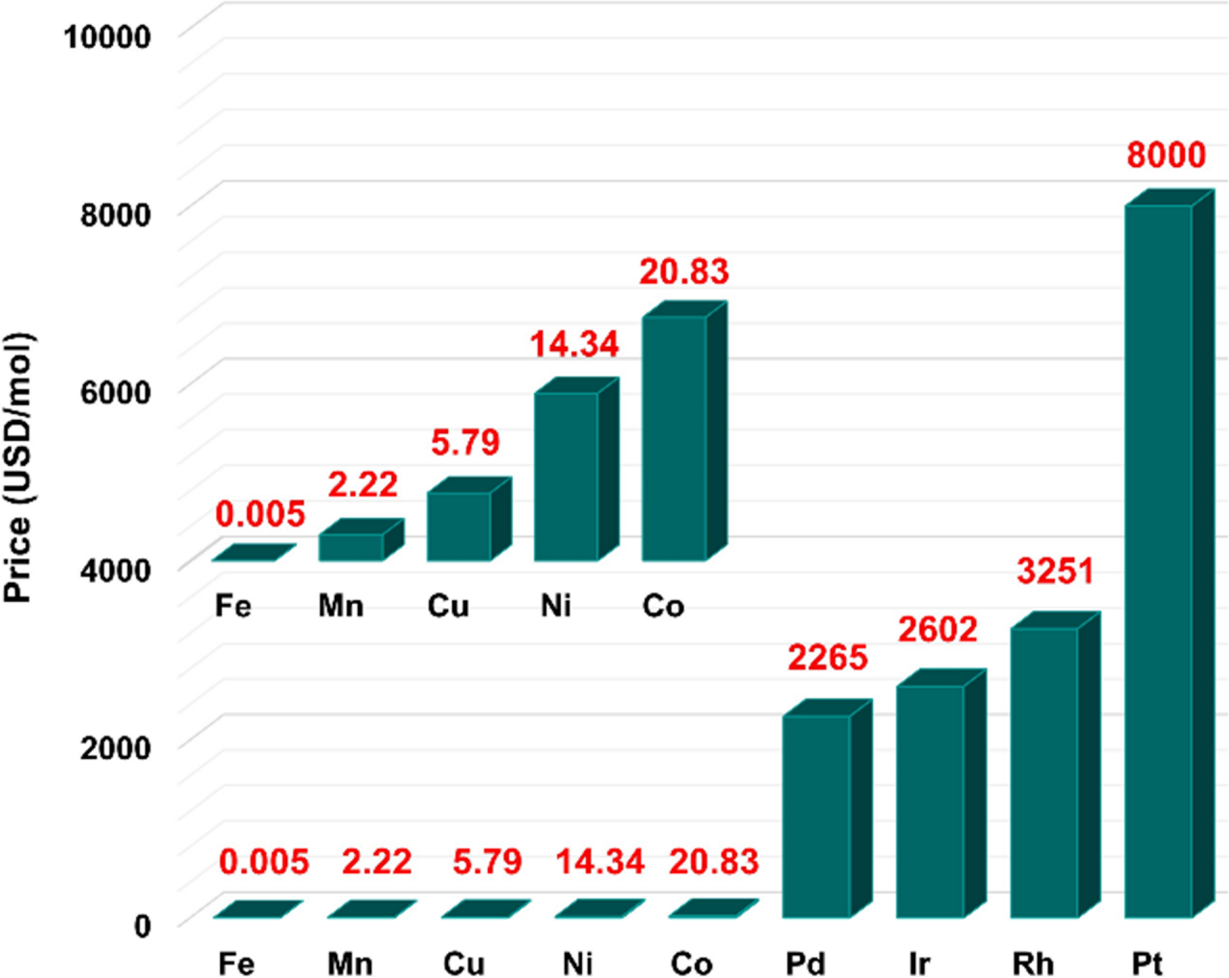
Price comparison among iron and other transition metals used in catalysis.

A prominent earth abundant TM bringing a renaissance to the idea of green catalysis is iron. Notably, iron is the most earth-abundant d-block element. Moreover, it is found iron is less expensive by several magnitudes compared to other late TM catalysts (Figure 1) [35].

Although the first Fe-catalyzed homo-coupling of aryl Grignard species was reported in 1941 by Kharasch and Fields [36], it wasn’t until 1971 Kochi and Tamura demonstrated the first Fe-catalyzed cross-coupling reaction between Grignard reagents and vinyl halides [37]. As of late, the development of Fe-catalyzed cross-coupling methodology and mechanistic rationales have burgeoned [38]. Today, the rate of growth within the field of iron catalysis is much greater than that compared to the more studied late TMs [39–43].

**Scheme 1:**
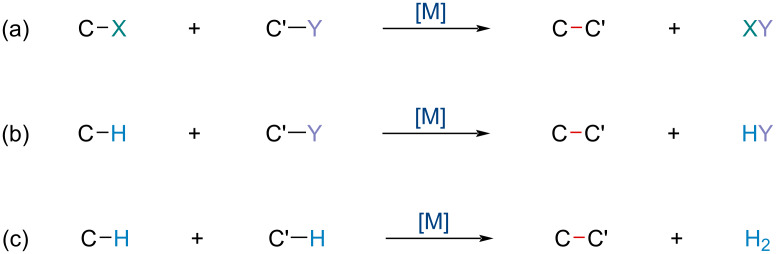
Typical modes of C–C bond formation.

Besides the more recognized concept of TM cross-coupling reactions revolving around an organic electrophile bearing a leaving group and an organometallic nucleophile, there is another large area of cross-coupling reactions that have been under significant development over the past 10 years. First achieved by Li and co-workers in 2007 [44], cross-dehydrogenative-coupling (CDC) reactions offer a highly atom economic approach to carbon–carbon (C–C) and carbon–heteroatom (C–X) bond formation via C–H activation [45–46]. Generally speaking, C–C bond forming reactions can be classified into three types (Scheme 1): the reaction of a compound bearing functional group (X), coupling with another compound bearing functional group (Y), producing a new C–C bond through the formation of X–Y (Scheme 1a). Secondly, the reaction of a C–H compound with a C–X functionalized compound (Scheme 1b). Lastly, the reaction between two C–H compounds to form a C–C bond, formally eliminating H_2_, hence the dehydrogenative reference (Scheme 1c). As this coupling reaction does not require functionalization prior to coupling, it shortens the synthetic route, and lowers the production of byproducts.

**Scheme 2:**
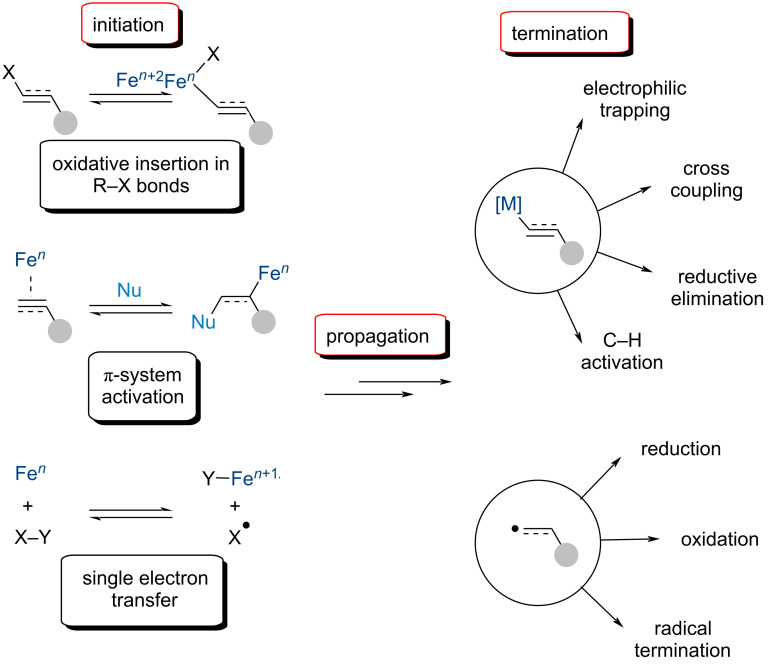
The components of an iron-catalyzed domino reaction.

Iron catalysis offers an attractive, and sustainable, approach to the aforementioned economic and ecological concerns. In the same vein, cascade reactions are important tools to meet such challenges currently facing synthetic chemists and have received considerable attention as of late. Introduced by Tietze, cascade reactions are sequences of transformations where subsequent transformations occur only in virtue of functionality formed in previous steps [47]. This process repeats until a product stable under the reaction conditions is formed and the reaction terminates. Compared to the late TMs, iron can possess a wide array of oxidation states, ranging from 2− to 6+, allowing for iron catalysis to be utilized and perform several different types of reactions. As such, a closer look at Fe-catalyzed cascade reactions reveals several distinct features. One possible method for the initiation of a multistep reaction is through the generation of an organoiron species. This can occur by the oxidative insertion of a low-valent iron into a C–X bond (Scheme 2). Evidently, iron in low oxidation states may operate as an iron-centered nucleophile, and catalyze reactions involving oxidative addition, transmetallation, and reductive elimination processes. On the other hand, iron may act as a Lewis acid, activating carbon–carbon multiple bonds via π-binding or heteroatoms via σ-complexes. This can either generate the organoiron complex after nucleophilic attack or produce a carbocation which will react further. However, reactions catalyzed by the Lewis-acidic character of iron salts are beyond the scope of this review. Iron also has the ability to transfer one or two electrons to a substrate. This opens the possibility for radical reactions via a single electron transfer (SET). Once initiated, the reaction will propagate, which typically involves the insertion of a π-system (carbometallation of alkenes/alkynes) in the case of organoiron species (Scheme 2). Alternatively, the generated radical species may undergo radical addition to alkenes, alkynes, or aromatic arenes. The final step is the termination of the reaction through the trapping of the reactive intermediate. Organoiron complexes have been shown to undergo electrophilic trapping with external species or proceed through cross-coupling eventually undergoing reductive elimination. Radical addition will typically conclude with the reductive addition or difunctionalization of the π-system; however, it has been demonstrated the radical intermediate can go through a SET oxidation/elimination to recover the initiating π-functionality.

In this review, Fe-catalyzed domino coupling reactions involving π-systems will be discussed. Recent methods in the pursuit for efficient and economical carbon–carbon and carbon–heteroatom bond-forming reactions, such as cross-coupling, CDC, and oxidative coupling/difunctionalizations, will be summarized. The review is categorized by reaction type, and the type of bonds being formed. For reasons of clarity, newly formed bonds are sketched in red, with newly formed cyclic structures being highlighted.

## Review

### Iron-catalyzed cross-coupling

Metal-catalyzed cross-coupling reactions have become a staple for carbon–carbon bond formation. The late TMs that have dominated the field of cross-coupling reactions have largely been relegated to coupling partners containing either sp^2^- or sp-hybridized carbons. Excellent progress has been made demonstrating reactions involving sp^3^-hybridized substrates, as well as systems bearing β-hydrogens, operate efficiently under certain Fe-catalyzed conditions constituting serious competition for the established late TM-catalyzed systems. For more information, covering non-sequential Fe-catalyzed cross coupling reactions, we direct the interested readers to the several excellent reviews that described the chemistry therein [48–51].

**Scheme 3:**
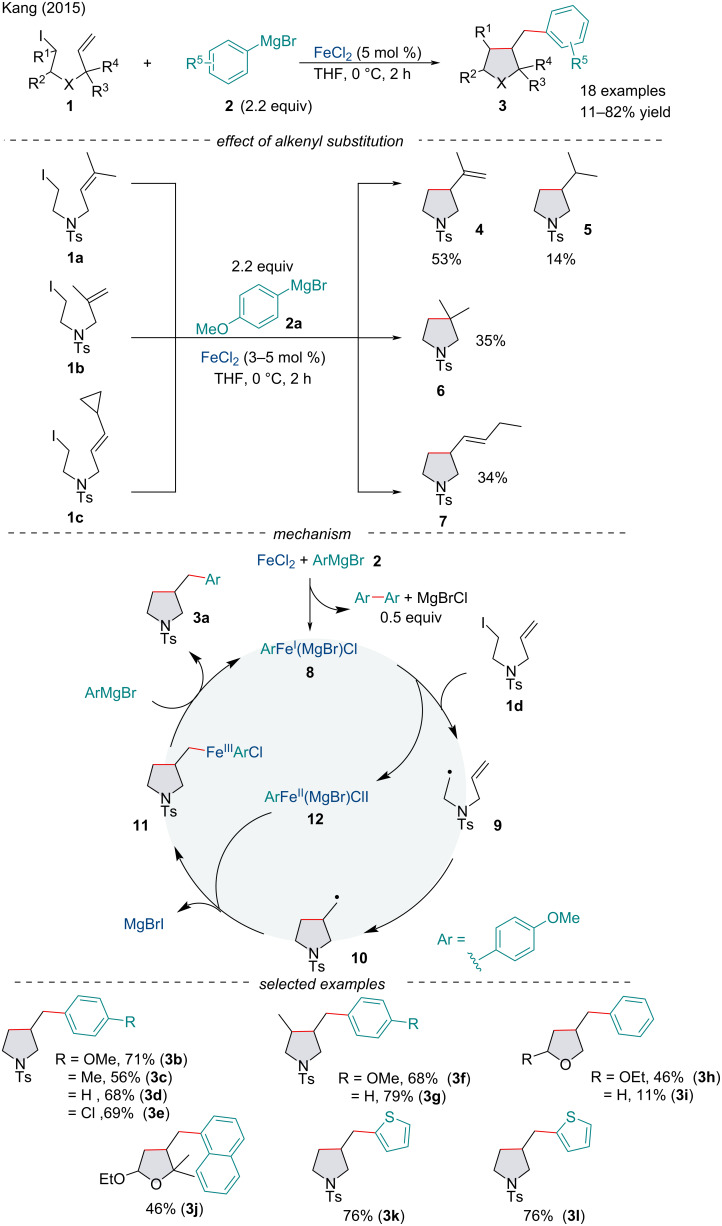
Iron-catalyzed tandem cyclization and cross-coupling reactions of iodoalkanes **1** with aryl Grignard reagents **2**.

The area of iron-catalyzed cross-coupling reactions of alkyl halides began in 2004 when Nakamura first reported the TMEDA-mediated Fe-catalyzed cross-coupling reaction between secondary bromides with aryl Grignard reagents [52]. Since then, several reports of alkyl halide cross-coupling reactions have been reported [53]. In 2015, Kang and co-workers described a FeCl_2_-catalyzed tandem cyclization/cross-coupling reaction of alkyl iodides **1** with aryl Grignard reagents **2** to give arylmethyl-substituted pyrrolidines and tetrahydrofurans **3** in poor to excellent yield (Scheme 3) [54].

The concept of alkyl halide tandem cross-coupling reactions was first introduced by Fürstner in 2004 who disclosed specific iodoalkanes with pendant olefins undergo cyclization prior to the anticipated cross-coupling with Grignard reagents [55]. This has since been recognized by a number of other reports, indicating a SET process [56–58]. The authors investigated the effect of alkenyl substitution on the reaction to better understand mechanistic details. On inspection of the results, it is clear the radical cyclization pathway precedes the cross-coupling pathway. Moreover, no dehalogenation or β-hydride elimination byproducts were detected which supports the absence of an initial oxidative addition between the alkyl iodide and the active Fe catalyst. The possibility of a radical process rather than ionic cross-coupling is supported by the tandem cyclization/cyclopropyl ring-opening reaction, similar to previous reports [59].

The authors proposed a plausible catalytic cycle based on a series of mechanistic studies (Scheme 3). First, FeCl_2_ will react with the aryl Grignard reagent to form an aryliron complex **8** which can undergo a SET with the iodoalkane to yield the radical substrate **9**. A 5-*exo-dig* cyclization will produce the pyrrolidinyl methyl radical **10** which may add to the iron center to form the Fe(III) complex **11**. Reductive elimination would give rise to the final product, and transmetallation with a Grignard reagent regenerates the active Fe species. Alternatively, release of the aryl radical via *ipso*-attack of the alkyl radical generating the cross-coupled product cannot be ruled out [57,60].

**Scheme 4:**
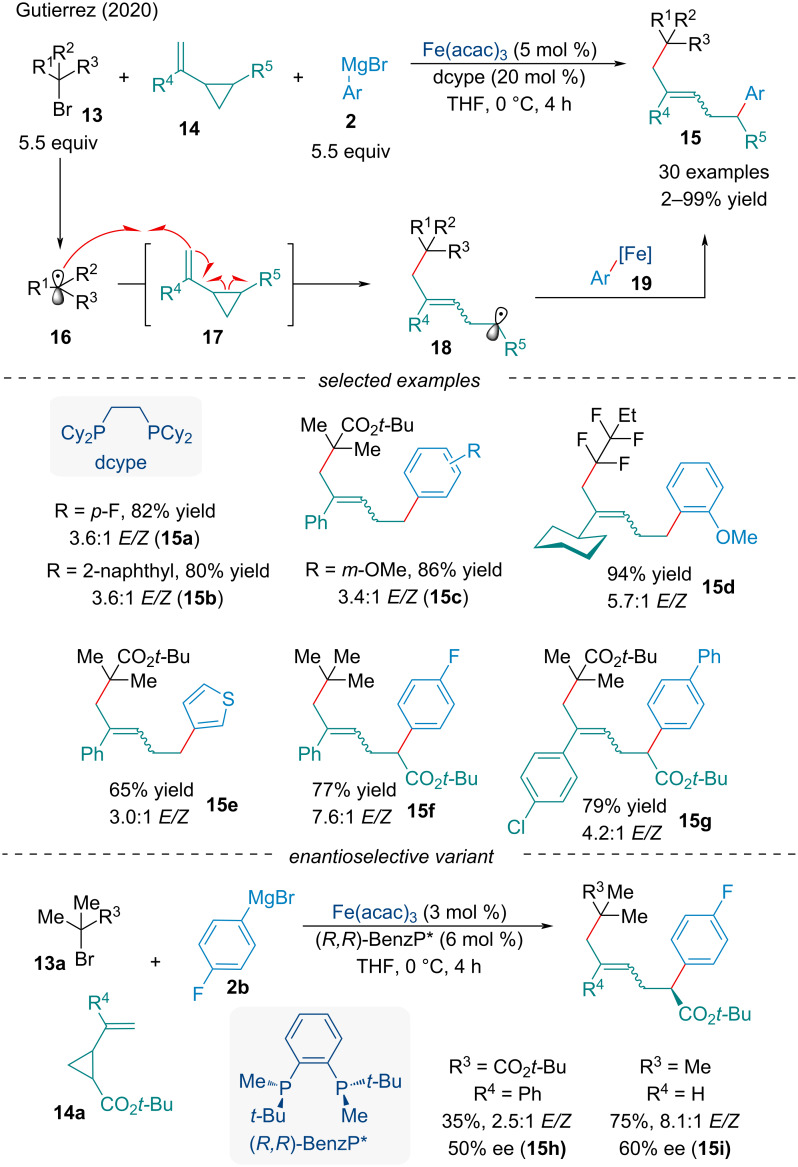
Three component iron-catalyzed dicarbofunctionalization of vinyl cyclopropanes **14**.

In 2020, Gutierrez and co-workers developed a Fe-catalyzed intra- and intermolecular difunctionalization of vinyl cyclopropanes **14** with alkyl bromides **13** and aryl Grignard reagents **2** (Scheme 4) [61]. Using sterically hindered tertiary alkyl bromides, the authors were able to favor intermolecular radical addition of the generated alkyl radical **17** to the vinylcyclopropane, outcompeting radical rebound to an aryl Fe species. The incipient radical can then undergo ring-opening of the cyclopropane **18**. Work by Fürstner [62] and Plietker [63] showed iron catalysts were able to ring-open vinylcyclopropanes for monocarbofunctionalization terminating in protonation; however, Gutierrez demonstrated the resultant radical could undergo Fe-catalyzed cross-coupling reactions. The authors noted the reaction was tolerable of both electron-donating and withdrawing groups on all three components of the reaction affording products in good yield; however, the reaction produced geometric isomers, consistently favoring the *E* isomer. The authors applied their methodology towards an asymmetric variant using a chiral diphosphine ligand. Preliminary results demonstrated the chiral iron species moderately controlled the enantioselectivity of the aryl Grignard cross-coupling. This work provided a proof-of-concept towards the use of vinylcyclopropanes as useful 1,5-synthons in asymmetric Fe-catalyzed cross-coupling reactions. Although poor enantioinduction was observed, several Fe-catalyzed non-sequential cross-coupling protocols have been established with yields and enantioselectivity rivaling Pd-catalyzed reactions [64–65]. Mechanistically, these reactions differ by not including a π-system which allows for propagation of reaction.

**Scheme 5:**
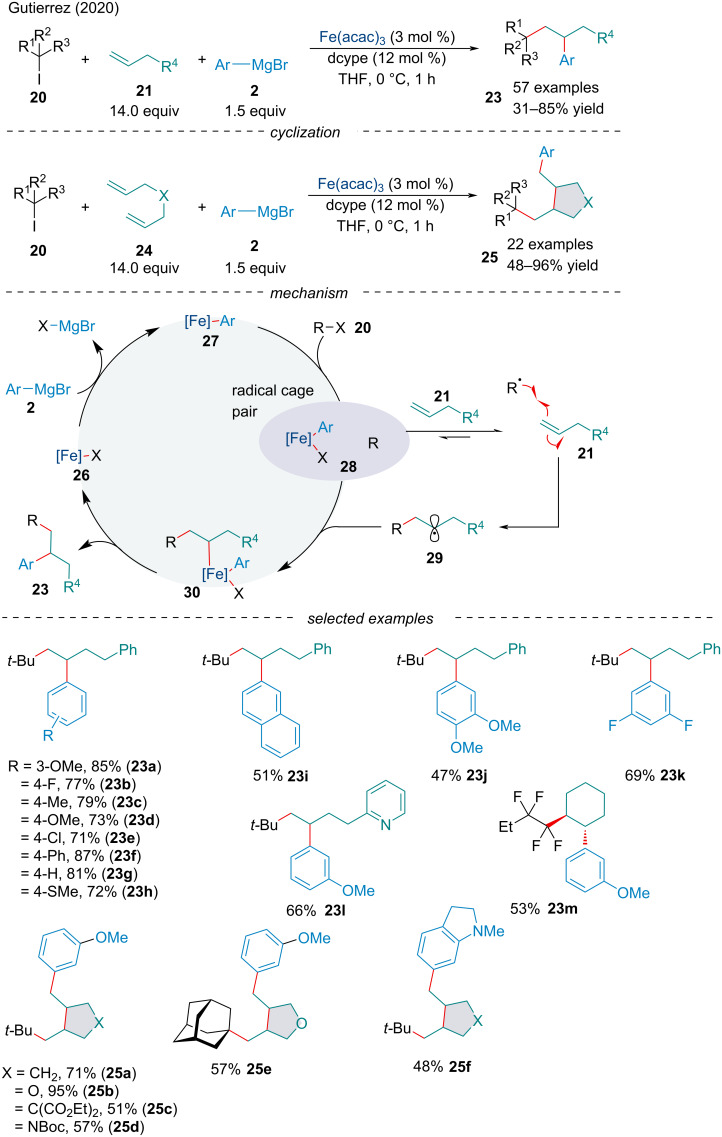
Three-component iron-catalyzed dicarbofunctionalization of alkenes **21**.

In the same year, the Gutierrez Lab reported the first three-component 1,2-dicarbofunctionalzation of alkenes **21** (Scheme 5) [66]. The authors noted π-systems bearing O- and S-heteroatoms had little to no compatibility with the transformation as well as sterically hindered Grignard reagents **2**. Similar to their previous report, primary and secondary alkyl halides were prone to undergoing direct cross-coupling rather than radical addition across the π-system. Consistent with the proposed mechanism, perfluorinated *n*-alkyl radicals performed well, suggesting ease of Giese addition is crucial [67]. The group expanded the reaction to include 1,6-dienes **24** leading to **25** via the formation of three C–C bonds through a radical cyclization/arylation cascade, like that reported by Kang et al. (Scheme 3).

The authors hypothesized the alkyl halide could react with aryl iron species **27** to form the alkyl radical **28** (Scheme 5). Regioselective Giese addition to the π-system **21** would generate the transient 2° alkyl radical **29**. Due to the high energetic barrier associated with direct cross-coupling between sterically hindered 3° alkyl radicals and aryliron complexes, it is assumed the persistent aryliron species is stable enough to be selectively trapped by the less sterically demanding 2° alkyl radical **29**. Reductive elimination would form the difunctionalized product and transmetallation with an aryl Grignard reagent regenerates the active Fe species **26**, restarting the catalytic cycle. As driving Giese addition is paramount, this method is currently limited to the use of a large excess of olefins; however, activated alkenes could circumvent this requirement.

**Scheme 6:**
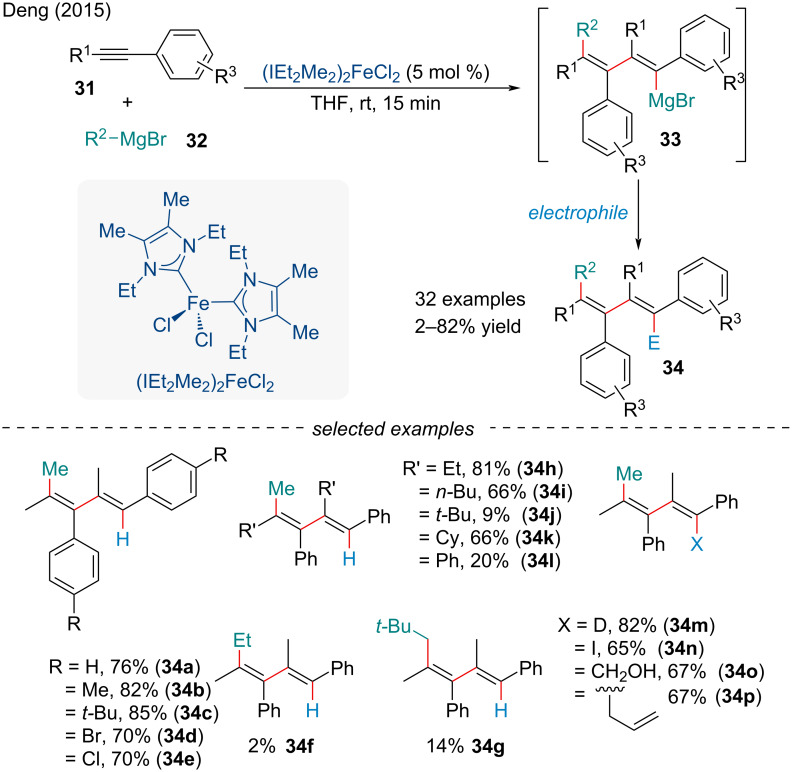
Double carbomagnesiation of internal alkynes **31** with alkyl Grignard reagents **32**.

In 2016, the Deng group studied a novel double carbomagnesiation of unsymmetrical internal alkynes **31** with alkyl Grignard reagents **32** producing 1,3-dienylmagnesium reagents **33** with high regio- and stereoselectivity (Scheme 6) [68]. A major problem with the carbomagnesiation of internal alkynes bearing no heteroatoms is the relatively harsh conditions required producing poor selectivity in some cases [69–70]. The strong σ-donating nature of the IEt_2_Me_2_NHC ligand and its appropriate steric properties are thought to be crucial to the success of the reaction [71]. The authors showed the in situ-formed 1,3-dienylmagnesium species **33** can also be trapped by a variety of electrophiles, demonstrating the synthetic utility of the reaction.

**Scheme 7:**
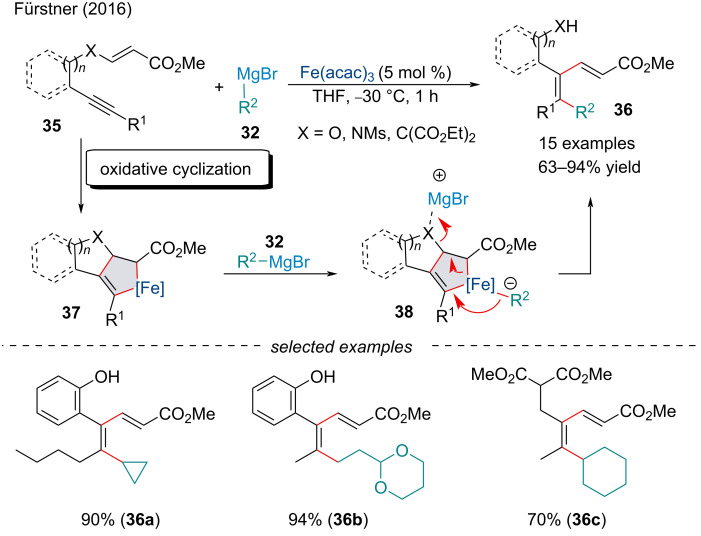
Iron-catalyzed cycloisomerization/cross-coupling of enyne derivatives **35** with alkyl Grignard reagents **32**.

In 2016, Fürstner and Echeverria demonstrated a mechanistically distinct protocol for the synthesis of 1,3-dienes **36** (Scheme 7) [72]. Compared to previous Fe-catalyzed carbomagnesiation reactions (Scheme 6) where carbometallation occurs in a concerted *syn*-manner this protocol, instead, is initiated by the oxidative cyclization of the 1,6-enyne **35** followed by reductive elimination of the carbon nucleophile **38**. Interestingly, this reaction proceeds via the cleavage of heteroelements and activated C–C bonds prior to reductive elimination of the metallacyclic ate-complex, resulting in the net formation of two new C–C bonds. Noteworthy, this methodology demonstrated a wide substrate scope, namely reacting smoothly with all-carbon backboned substrates **36c**, as well as being applicable to esters and tosylamides, proving it to be a powerful protocol for the synthesis of stereocontrolled tetrasubstituted alkenes.

**Scheme 8:**
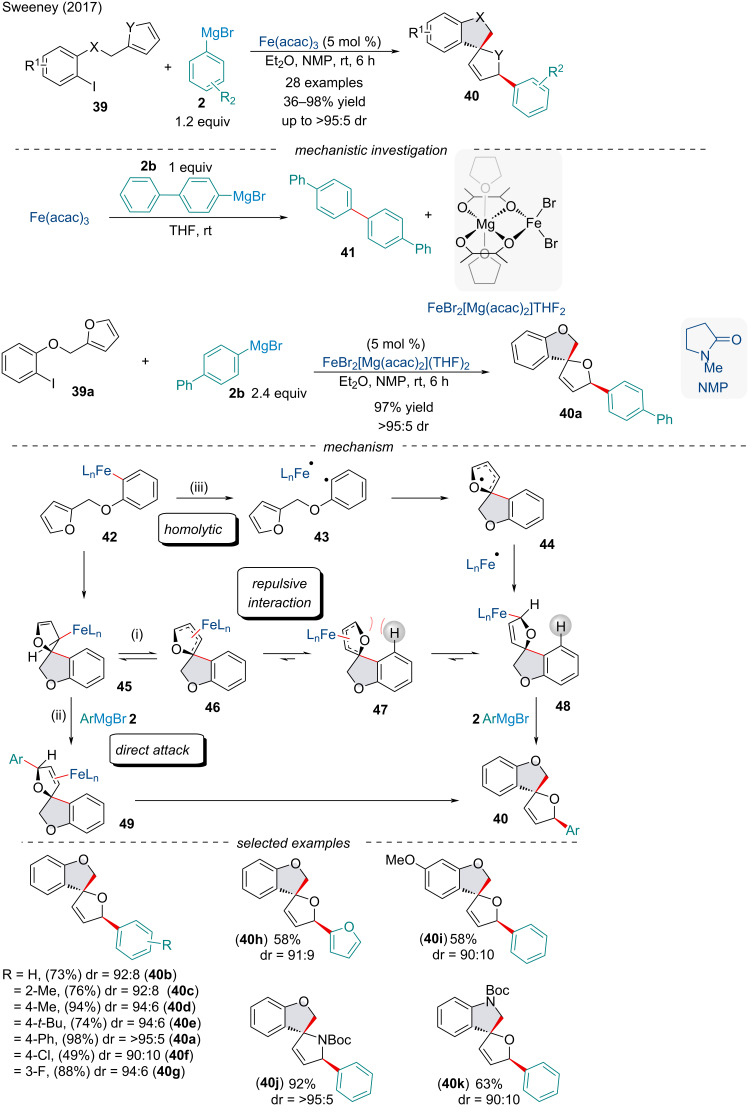
Iron-catalyzed spirocyclization/cross-coupling cascade.

In 2017, Sweeney and co-workers established a Heck/Kumada cross-coupling cascade to construct nitrogen and oxygen-containing *cis*-heterospirocycles **40** in high yield and diastereoselectivity with inexpensive Fe(acac)_3_ as the precatalyst (Scheme 8) [73]. Interestingly, this protocol was applicable to substrates bearing classically sensitive functionalities like esters and aryl chlorides. Exposure of the iron catalyst to one equivalent of aryl Grignard reagent **2b** in the absence of the halide substrate afforded the bimetallic Fe(II) complex FeBr_2_[Mg(acac)_2_](THF)_2_. Using FeBr_2_[Mg(acac)_2_](THF)_2_ in place of Fe(acac)_3_ in the arylative spirocyclization reaction delivered product **40a** in comparable yield, suggesting an initial in situ reduction of the Fe(III) precatalyst occurs in the early stages of the catalytic cycle.

Although the mechanisms of Fe-catalyzed cross-coupling reactions are often complex [74–75] (Scheme 8), the authors believe this reaction likely proceeds with the σ-aryliron intermediate **42** cyclizing to give the η^1^-allyliron species **45**. Isomerization of **45** would deliver the less-hindered isomer **48** (path i). The stereochemical outcome can be rationalized by the steric interactions of the iron residue and the C–H bond of the aromatic ring in **47**. Capturing of the iron complex by the Grignard reagent **2**, followed by reductive elimination would deliver the observed product **40**. Alternatively, the iron species **45** may undergo direct *anti*-attack by the Grignard reagent (path ii) [76]. One final possibility is the reaction proceeds via a radical mechanism (path iii) [77], although use of radical inhibitors had little impact on the success of the reaction. It seems unlikely a radical pathway is involved in the reaction mechanism; however, it cannot be categorically excluded.

**Scheme 9:**
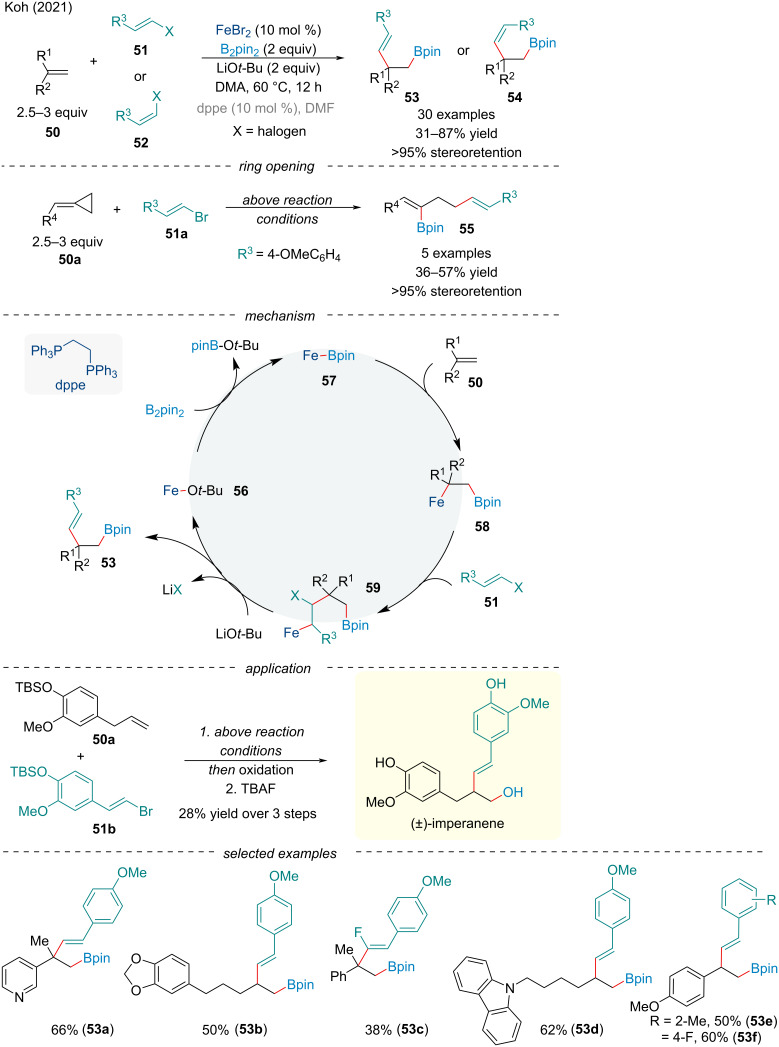
Iron-catalyzed alkenylboration of alkenes **50**.

In 2021, the Koh group demonstrated the first three-component alkenylboration of alkenes **50** (Scheme 9) [78]. The authors noted the described methodology regioselectively installs both the boryl functionality and olefin across both activated and unactivated π-systems **50**; however, the later required the use of (dppe)FeBr_2_ in DMF to deliver products in appreciable yield. Alkenyl fluoride, chloride, and bromide substrates **51**/**52** were found to be amenable to the reaction although with varying degrees of success, likely due to the competing base-promoted 1,2-elimination. With the cyclopropylidene-functionalized substrates **50a**, ring-cleavage led to trisubstituted (*E*)-alkenylboronates **55**, acting as a 1,5-synthon, similar to Gutierrez‘s vinylcyclopropanes (Scheme 4) [66]. Based on mechanistic investigations, Koh proposed the catalytically active iron-boryl species **57** is generated through the ligand exchange of **56** with B_2_pin_2_ which can undergo borylmetallation across the alkene in a *syn*-fashion **58**. Side-on coordination of the haloalkene’s π-bond can trigger a *syn*-carbometallation **59**. A base-mediated 1,2- elimination will deliver the alkenylboration product as well as regenerate **56**. The methodology was applied towards the synthesis of (±)-imperanene.

### Iron-catalyzed cross dehydrogenative coupling

Transition-metal-catalyzed carbon–carbon (C–C) or carbon–heteroatom (C–X) bond formation involving two different C–H bonds or one C–H and one X–H bond is formally known as cross dehydrogenative coupling (CDC) and is quite attractive to synthetic organic chemists [79]. Such coupling eliminates the need for prefunctionalization of the substrate, thus making synthetic schemes shorter and more efficient improving the atom and step-economy of the reaction. Other than the clear economic benefits CDC offers, it’s also a facile method for the coupling of sp^3^ C–H species. However, the CDC reaction is not without its challenges, mainly due to the poor reactivity of C–H bonds; thus, chemists have devised protocols to activate different types of C–H bonds for the formation of C–C and C–X bonds. We classified the different CDC cascade reactions into two different sections: strictly carbon CDC reactions and heteroatomic CDC reactions.

#### Iron-catalyzed carbon–carbon cross dehydrogenative coupling

**Scheme 10:**
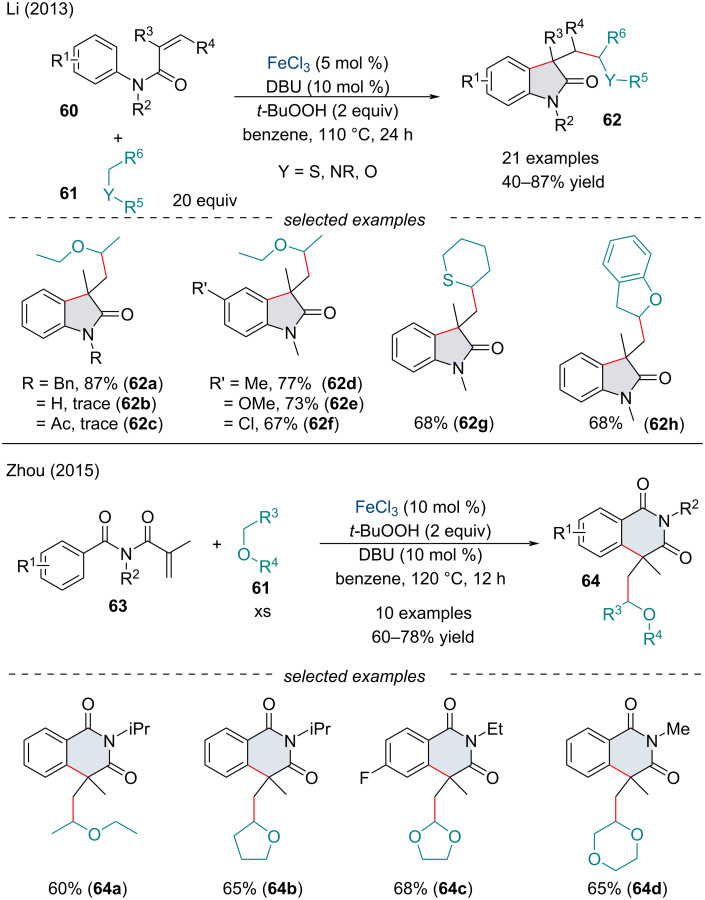
*N*-Alkyl–*N*-aryl acrylamide **60** CDC cyclization with C(sp^3^)–H bonds adjacent to a heteroatom.

In 2013, Li and co-workers reported the FeCl_3_-catalyzed arylalkylation of activated alkenes **60** for the synthesis of oxindoles **62** (Scheme 10) [80]. Mechanistic studies, including kinetic isotope effects and radical trapping, suggested a radical mechanism. The hydroperoxide, in the presence of an iron catalyst, abstracts the hydrogen atom alpha to the heteroatom. The alkyl radical may attack the acrylamide; subsequent intramolecular radical cyclization with the aryl ring would give the oxindole scaffold. Hydrogen abstraction would regenerate the reduced iron catalyst and produce the final product. Two years later, Zhou and co-workers expanded the reaction for the synthesis of substituted isoquinoline-1,3(2*H*,4*H*)-dione derivatives **64** (Scheme 10) [81]. Both laboratories observed similar trends in reactivity and came to the same mechanistic conclusions. Mechanistically, sequential and nonsequential CDC reactions are nearly identical. Typically, a CDC reaction is initiated through iron-mediated oxidation processes. In the case of a sp^3^ C–H species, an alkyl radical can be generated. This is where sequential and nonsequential CDC reactions diverge. In the case of a nonsequential CDC reaction, the alkyl radical will directly attack an electrophilic species [79]. On the other hand, sequential CDC reactions involve propagation reactions. These propagation steps typically involve a radical addition across a two-carbon fragment, generating a new carbon-based radical species. There is no limit to the number of propagation sites a coupling partner can have; however, controlling the chronology of the radical additions can be difficult. Once the intermediate has expended all propagation sites it is terminated, typically through the generation of a new radical species.

The Zhu group followed up on this work by disclosing the use of acetonitrile as the radical precursor for the cyanomethylation/arylation of arylacrylamides to access oxindoles [82]. Despite the small scope of aliphatic nitriles explored, the reaction further demonstrated the synthetic potential of C(sp^3^)–H species within CDC methodology.

**Scheme 11:**
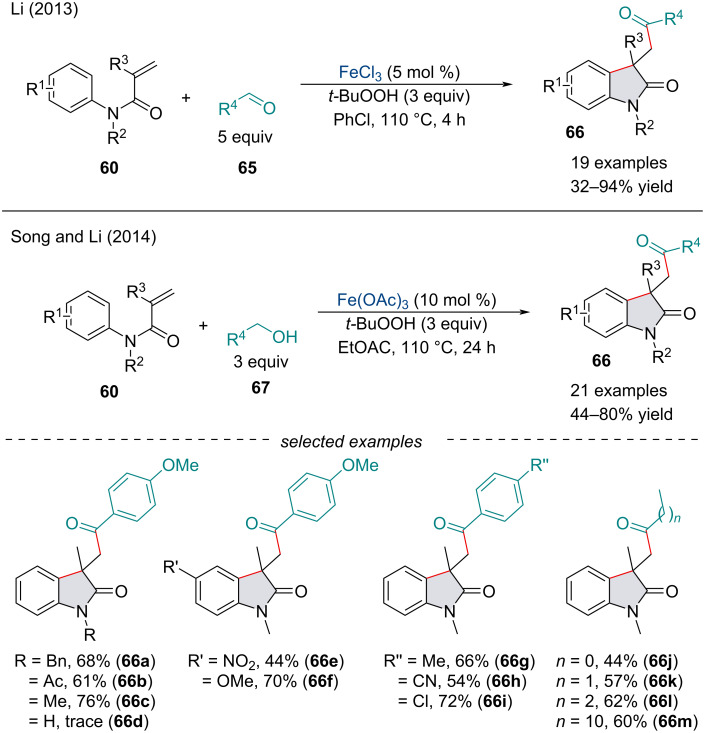
1,2-Carboacylation of activated alkenes **60** with aldehydes **65** and alcohols **67**.

In 2013, the Li group established a carbonyl-arylation of *N*-arylacrylamides **60** with alkyl and aryl aldehydes **65** (Scheme 11) [83]. Like Li’s report in 2013 (Scheme 10) [80], the reaction begins with a radical addition to the acrylamide **60** followed by subsequent radical cyclization with the aryl ring. A few substituent effects were noted, namely *ortho*-substituents on the aryl ring were detrimental to the reaction. Moreover, terminal alkenes preformed poorer than their 1,1-disubstituted counterparts, perhaps due to the generation of the more stable 3° radical intermediate. In the following year, Song and Li reported a reaction shortcut for the carbonyl-arylation of *N*-arylacrylamides **60** through the *in-situ* oxidation of alcohols **67** (Scheme 11) [84]. Under the optimized reaction conditions, both primary and secondary alcohols are oxidized to the corresponding aldehyde/ketone, so the chronology of the addition remains unclear whether the reaction proceeds exclusively via an alkyl radical followed by subsequent oxidation, an acyl radical, or a combination of both. Further, slight modifications of the reaction conditions have allowed for the synthesis of indolines and dihydropyran frameworks through tandem carbonylarylation and carbamoylarylation reactions of olefins [85–86].

**Scheme 12:**
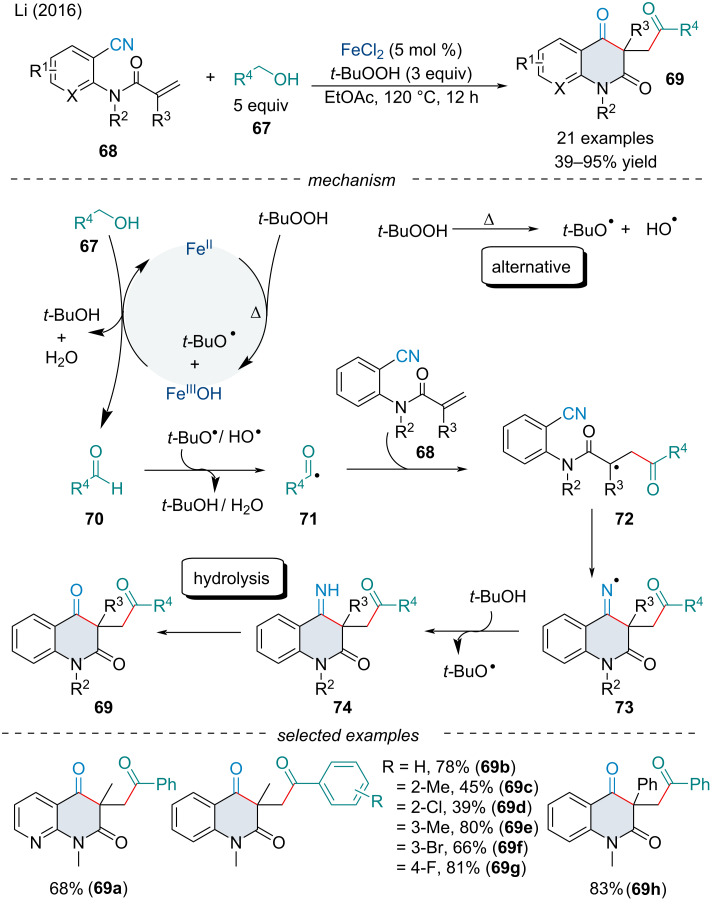
Iron-catalyzed dicarbonylation of activated alkenes **68** with alcohols **67**.

In 2016, Li and co-workers investigated the dicarbonylation of alkenes **68** (Scheme 12) [87]. It was noted both EWGs and EDGs on the phenyl ring were amenable to the reaction; however, the yield was dramatically reduced with electron-deficient *N*-substituents. Substitution of the alcohol partner was well-tolerated though sterically demanding functionality lowered its reactivity. On the basis of the experimental results, the authors proposed a catalytic cycle (Scheme 12). First, the hydroperoxide, in the presence of an Fe(II) species, generates an Fe(III) intermediate and the alkoxy radical which can oxidize the incoming alcohol **67** to an aldehyde **70**. Another equivalent of hydroxy radical, either generated under thermal conditions or through the Fe redox cycle, can abstract the aldehydic hydrogen to form the acyl radical **71**. Subsequent radical addition to the alkene **68** to form **72** followed by cyclization with the nitrile affords the iminyl radical **73** which can abstract a hydrogen atom to form the more stable imine **74**. Hydrolysis of the imine affords the final product **69**. In 2020, Sun and Liu reported the iminyl cyclization could also be achieved with DMSO as a methyl-radical precursor [88].

**Scheme 13:**
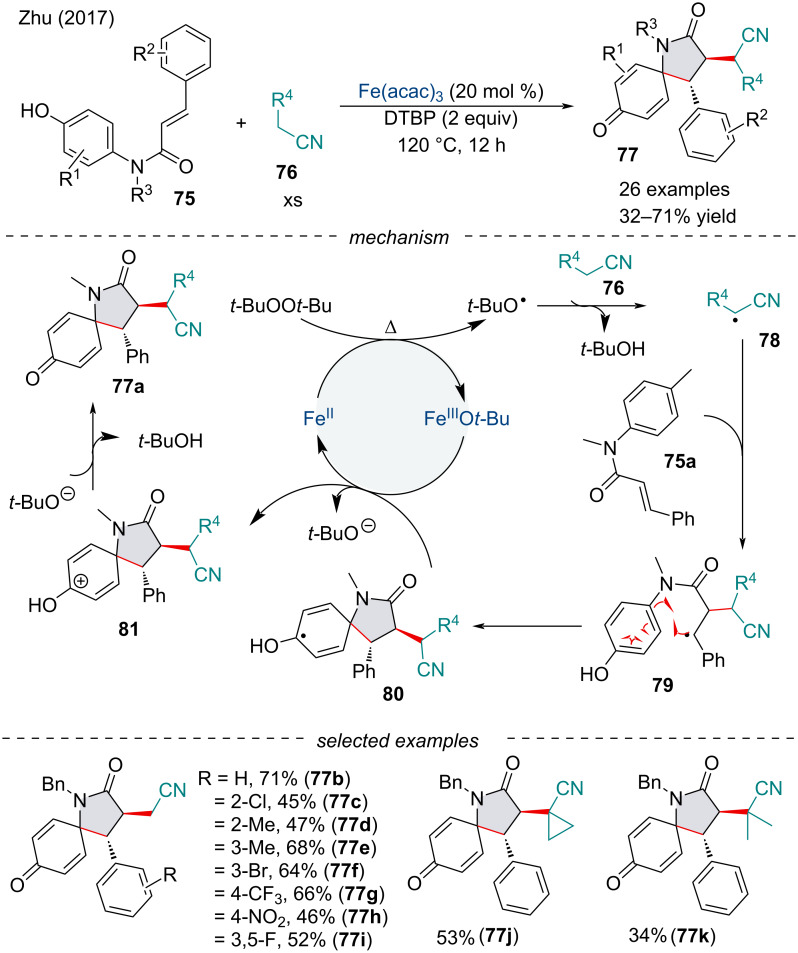
Iron-catalyzed cyanoalkylation/radical dearomatization of acrylamides **75**.

In 2017, the Zhu group developed an Fe(acac)_3_-catalyzed cyanoalkylative dearomatization of *N*-phenylcinnamamides **75** for the synthesis of 1-azaspiro[4.5]decanes **77** (Scheme 13) [89]. The reaction was amenable to both EWGs and EDGs; however, substitution at the *ortho*-position of the cinnamamide lowered the product yield. Mechanistic experiments suggest the reaction proceeds through a radical reaction. Moreover, kinetic isotope studies revealed the cleavage of the C(sp^3^)–H bond may be involved in the rate-determining step of this transformation. Mechanistically, prototypical homolysis of the peroxide in the presence of the Fe(II) catalyst will generate the alkyl radical **78** formed via hydrogen abstraction. The intermediate **78** may regioselectively attack the α-position of the carbonyl **75a**. A thermodynamically controlled 5-*exo* cyclization with the aryl ring **79** would afford the spirocyclic intermediate **80**. The authors theorize the >20:1 diastereoselectivity of the reaction arises from the steric interaction between the aromatic and cyano groups. The oxidation of species **80** would reduce the Fe catalyst back to its reduced form, while a *tert*-butyl alkoxide can furnish the final product through acid-base catalysis.

**Scheme 14:**
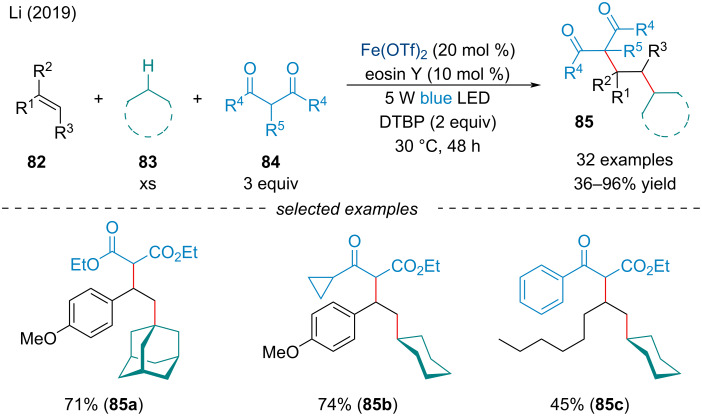
Synergistic photoredox/iron-catalyzed 1,2-dialkylation of alkenes **82** with common alkanes **83** and 1,3-dicarbonyl compounds **84**.

In 2019, Li and co-workers investigated a three-component dialkylation of alkenes **82** with common alkanes **83** and 1,3-dicarbonyl compounds **84** via synergistic photoredox/iron catalysis (Scheme 14) [90]. This protocol parallels Li’s seminal report in 2007 [44]; however, under these reaction conditions, the reactive radical was propagated across an alkene before termination with the activated methylene unit. Notably, the reaction did not proceed in the dark or in the absence of the photosensitizer at 30 °C; further, the reaction generated the desired product in lower yield at 120 °C. The scope was broad and tolerated a wide array of 1,3-keto esters and 1,3-diketones, as well as both benzylic and aliphatic C(sp^3^)–H compounds.

#### Iron-catalyzed heteroatomic cross dehydrogenative coupling

**Scheme 15:**
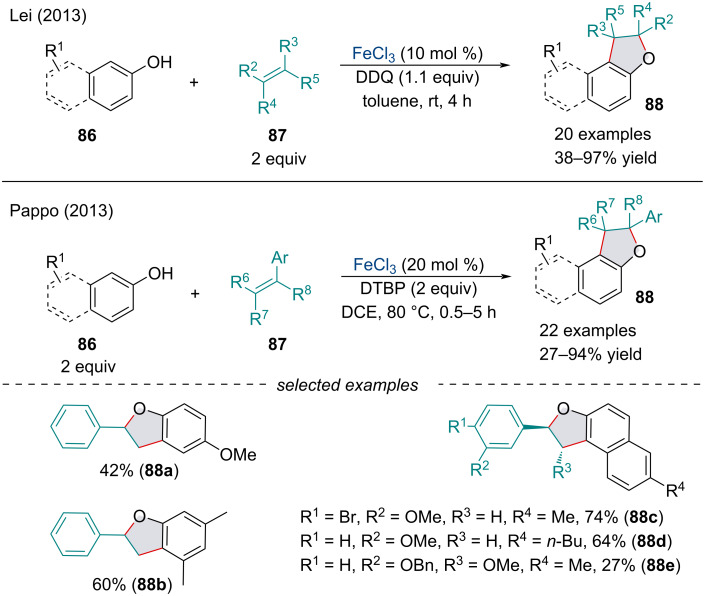
Iron-catalyzed oxidative coupling/cyclization of phenol derivatives **86** and alkenes **87**.

In 2013, Lei and Pappo independently reported an FeCl_3_-catalyzed oxidative coupling/cyclization cascade of phenol derivatives **86** and alkenes **87** (Scheme 15) [91–92]. Similar trends were reported by both groups namely electron-rich phenols, as well as those bearing halogen substituents, were suitable substrates under these reaction conditions. In Lei’s report, the reaction shuts down in the presence of TEMPO and in the absence of DDQ; thus, the formation of a phenoxy radical was proposed. In 2018, Zhong and co-workers reported a similar approach towards the assembly of 2,2-disubstituted indolines from *N*-sulfonylanilines and substituted styrene derivatives [93].

**Scheme 16:**
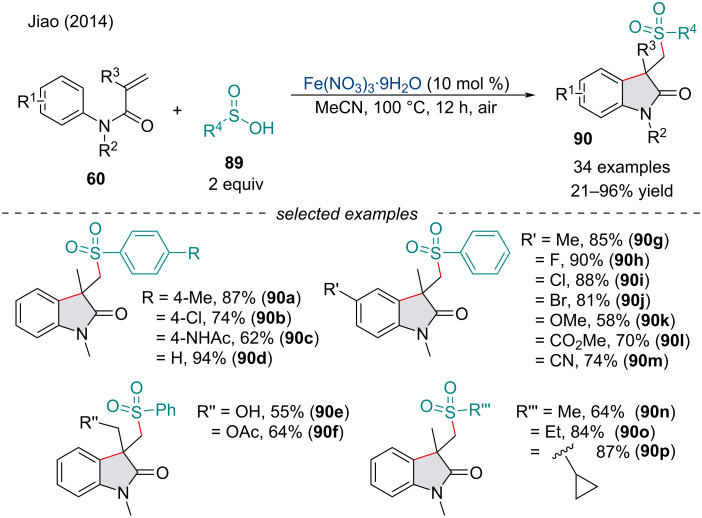
Iron-catalyzed carbosulfonylation of activated alkenes **60**.

In 2014, the Jiao group investigated the carbosulfonation of alkenes **60** for the synthesis of oxindoles **90** through sequential C–S/C–C-bond formation (Scheme 16) [94]. Interestingly, the protocol used air as the oxidant, avoiding the use of stoichiometric oxidants like previous radical cyclization cascades. Generally, substrates with an electron-withdrawing group afforded the product in greater yield. The reaction proceeds through the formation of a sulfonyl radical under aerobic conditions. Tandem attack on the alkenyl π-system with the sulfonyl radical followed by radical cyclization with the aryl ring constructs the substituted oxindoles **90**.

**Scheme 17:**
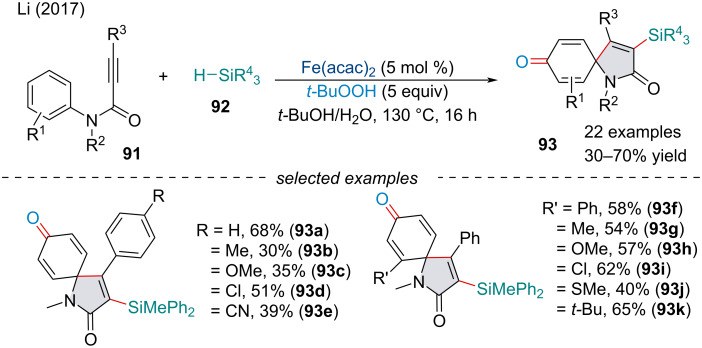
Iron-catalyzed oxidative spirocyclization of *N*-arylpropiolamides **91** with silanes **92** and *tert*-butyl hydroperoxide.

In 2017, Song and Li reported an oxidative spriocyclization of *N*‐arylpropiolamides **91** with silanes **92** for the synthesis of 3‐silylspiro[4,5]trienones **93** in good yield (Scheme 17) [95]. Compared to previously reported inter-/intramolecular CDC cascades, the authors were able to capture the post-cyclization aryl radical with the peroxide initiator rather than simply terminating the reaction with protonation. In terms of the scope of the reaction, substrates bearing an electron-rich functionality were less reactive than substrates with electron-deficient groups. Isotopic labeling revealed the oxygen functionality installed came from the peroxide initiator rather than the water present, suggesting the water plays another role in the reaction, potentially as a promoter of the catalyst.

**Scheme 18:**
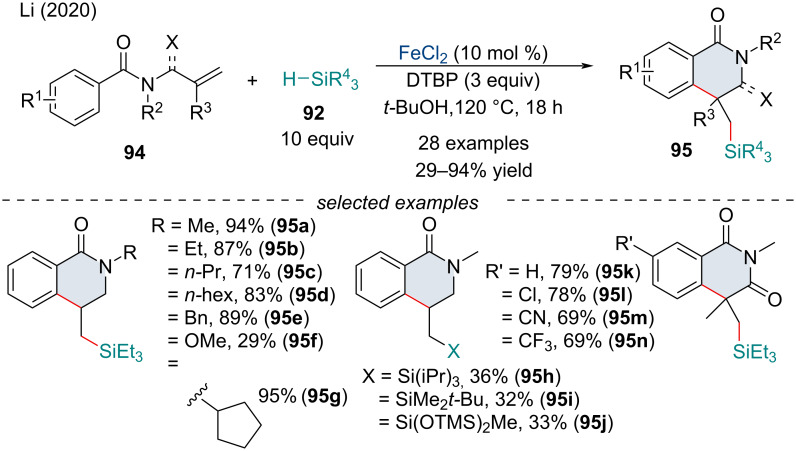
Iron-catalyzed free radical cascade difunctionalization of unsaturated benzamides **94** with silanes **92**.

In 2020, Li and co-workers investigated a carbosilylation cascade for the synthesis of various silylated dihydroisoquinolinones and 1,3-isoquinolinediones **95** (Scheme 18) [96]. The scope of the reaction was broad and could tolerate a variety of electron-donating and electron-withdrawing groups, though bulky silanes **92** afforded the products in reduced yield. The reaction proceeds through the formation of a silicon-centered radical generated via a Fe redox cycle (vide supra). Sequential attack on the alkenyl π-system followed by radical cyclization with the aryl ring constructs the 6-membered heterocyclic framework.

**Scheme 19:**
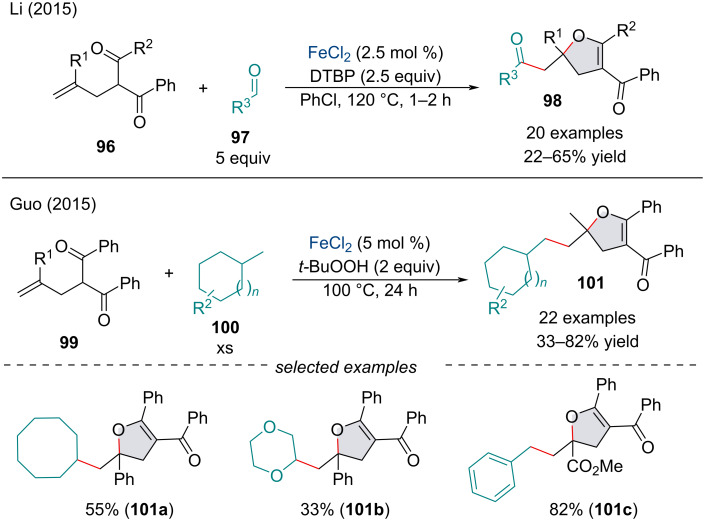
Iron-catalyzed cyclization of olefinic dicarbonyl compounds **97** and **100** with C(sp^3^)–H bonds.

In 2015, the Li group investigated the radical addition/cyclization of olefinic malonate and β-diketone compounds **96** with aldehydes **97** (Scheme 19) [97]. The reaction was feasible with ketones; however, lower product yields were observed. The efficiency of the reaction seems to be dependent on the deprotonation of the α-position of the olefinic malonate species. The authors noted decarbonylated products were obtained when cyclohexane carboxaldehyde and pivaldehyde were applied, consistent with the stability of the generated acyl radicals [98]. Concurrently, Guo and co-workers reported a similar approach towards the synthesis of dihydrofurans **101** through the sequential radical addition/cyclization of inactivated C(sp^3^)−H bonds **100** with olefinic dicarbonyl species **99** (Scheme 19) [99]. Both accounts found the reaction was shut down in the presence of radical scavengers. Both laboratories suggested the mechanism, wherein, after *tert*-butoxyl radical production, hydrogen abstraction can generate the appropriate radical species. Subsequent radical addition to the π-system followed by a 5-*endo*-trig radical cyclization would afford the furan framework. Following Fe-catalyzed oxidation, the resulting oxonium cation can be deprotonated to afford the final dihydrofuran. Further, this inter-/intramolecular radical addition/cyclization methodology has been applied for the synthesis of various substituted dihydropyrans [100–101] and dihydrofurans [102].

**Scheme 20:**
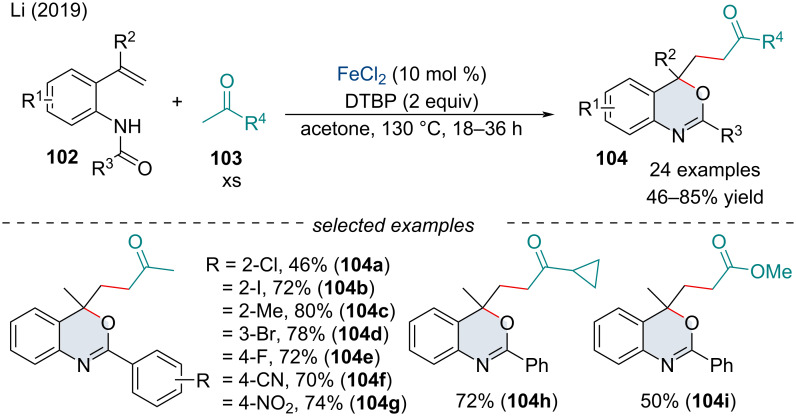
Radical difunctionalization of *o*-vinylanilides **102** with ketones and esters **103**.

In 2019, the Li group studied the selective activation of the α-C(sp^3^)–H of ketones and esters **103** for the tandem addition/cyclization of *o*-vinylanilides **102** (Scheme 20) [103]. Through a series of mechanistic experiments, it was noted the cleavage of the C(sp^3^)–H bond may be involved in the rate-determining step of this transformation, as well as free radicals being involved in the reaction mechanism.

**Scheme 21:**
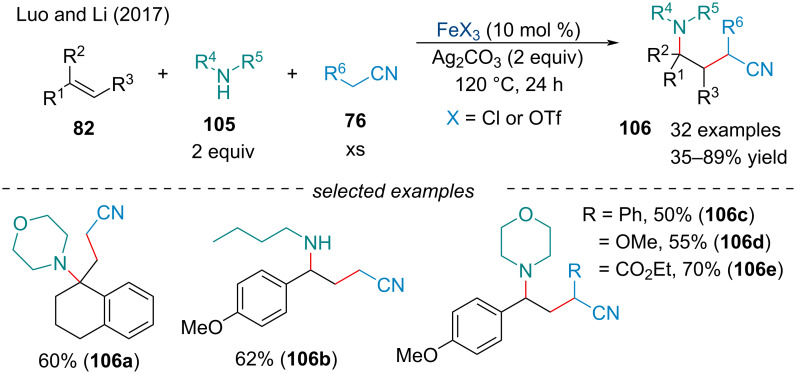
Dehydrogenative 1,2-carboamination of alkenes **82** with alkyl nitriles **76** and amines **105**.

In 2017, Luo and Li described a three-component Ag-mediated Fe-catalyzed 1,2-carboamination of alkenes **82** using alkyl nitriles **76** and amines **105** for the synthesis of γ-amino alkyl nitriles **106** (Scheme 21) [104]. The use of Ag_2_CO_3_ as a SET oxidant was shown to be key for the success of the reaction, as typical organic oxidants, like peroxides, displayed low activity. No clear trend was observed for the difference in efficiency between the Fe catalysts used. It was noted the use of the Fe catalyst wasn’t necessary to promote the reaction, but the yield of the cascade was significantly increased upon loading. The scope of the π-systems was limited to alkenes conjugated to electron-rich aryl species. Various nitrogen nucleophiles including primary/secondary amines and sulfonamides were compatible with the nucleophilic capture process. In the proposed mechanism, the α-hydrogen of the alkyl nitrile is deprotonated to form an organosilver species which undergoes SET oxidation with Ag(I) to afford the alkyl radical. Next, the α-cyanocarbon radical can add across the styrene derivative generating a benzylic radical which can be oxidized by Ag(I) to afford the corresponding benzylic cation. Nucleophilic trapping with an amine will produce the final product.

**Scheme 22:**
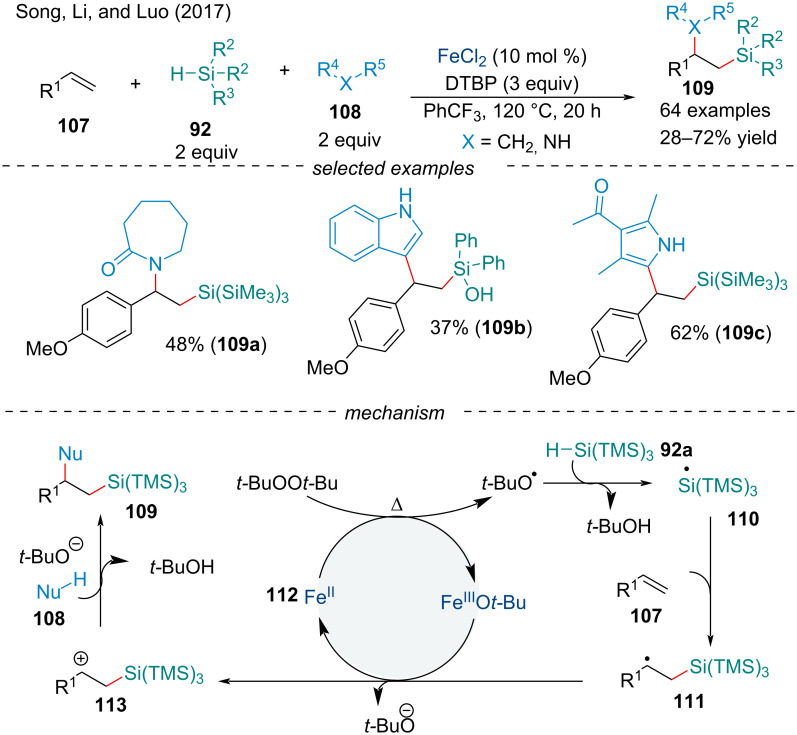
Iron-catalyzed intermolecular 1,2-difunctionalization of conjugated alkenes **107** with silanes **92** and nucleophiles **108**.

In the same year, Song and co-workers reported a dehydrogenative 1,2-difunctionalization of conjugated alkenes **107** with silanes **92** and various nucleophiles **108** (Scheme 22) [105]. This protocol offers an expedient approach to 1-amino-2-silylalkanes, a classically difficult framework to synthesize, typically requiring harsh reaction conditions, multistep synthetic routes, or the use of expensive silicon reagents [106]. Moreover, the methodology was extended to the carbosilylation of olefins with carbon nucleophiles **108** including indoles, pyrroles, and 1,3-dicarbonyls. The scope of the reaction was broad and could tolerate a variety of functional groups; however, electron-deficient alkenes afforded the products in slightly diminished yield. After the prototypical homolysis of the peroxide in the presence of the Fe(II) catalyst, a silicon-centered radical **110** is formed via hydrogen abstraction. The addition of radical **110** across the alkene generates the alkyl radical intermediate **111**. Oxidation of **111** by Fe(III)O*t*-Bu delivers the alkyl cation **113**. Nucleophilic trapping of the carbocation provides the final product. In 2018, the Li group continued to explore Fe-catalyzed silylation cascade chemistry. Their protocol investigated the silylperoxidation of activated alkenes in good yield [107].

**Scheme 23:**
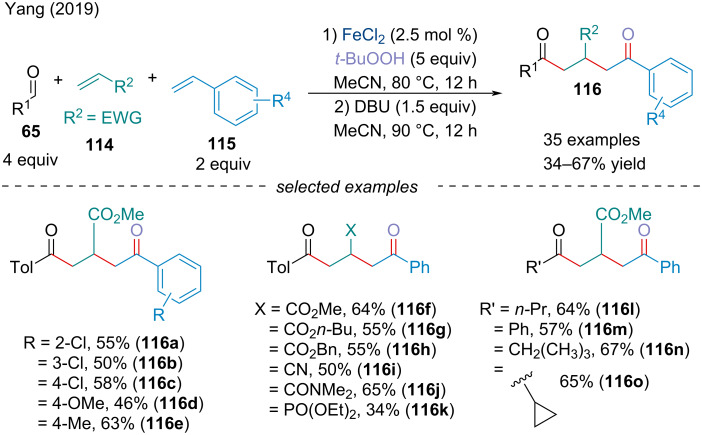
Four-component radical difunctionalization of chemically distinct alkenes **114**/**115** with aldehydes **65** and *tert*-butyl hydroperoxide.

In 2019, Yang and co-workers described a four-component radical dual difunctionalization and ordered assembly of two chemically distinct alkenes **114**/**115**, aldehyde **65**, and *tert*-butyl peroxide (Scheme 23) [108]. In order to selectively couple one alkene to another, without the formation of oligomers, the authors utilized the different electronic properties of the alkenes to control the chronology of the additions. The rate of addition of the acyl radical to an electro-deficient alkene is about three times greater than that of a styrene derivative [109–110]. The electrophilic radical, adjacent to an EWG, will favor the subsequent addition to the styrene derivative selectively to afford a metastable benzyl radical [111] which is captured by BuOO^•^ via a radical termination process.

### Iron-catalyzed oxidative addition/coupling and functionalization

#### Carbofunctionalization

Moving forward, the Fe-catalyzed carbofunctionalization of alkenes will be discussed as a method for the formation of multiple bonds in a single step. Mechanistically, Fe-catalyzed oxidative addition and functionalization reactions proceed similarly to cross dehydrogenative couplings (vide supra); however, these reactions will result in byproducts other than the formal elimination of H_2_. This section is categorized by the initiating step and the types of bonds being formed.

#### Denitrogenative C–C/C–C coupling

**Scheme 24:**
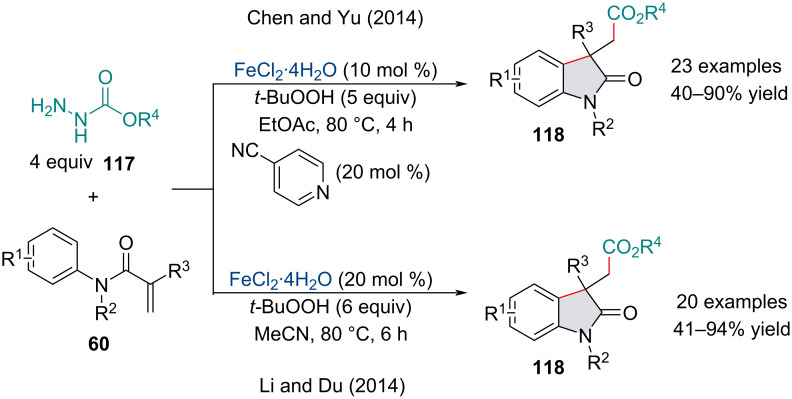
Iron-catalyzed carbocarbonylation of activated alkenes **60** with carbazates **117**.

In 2014, Yu’s and Du’s groups independently described the arylcarbonylation of alkenes for the synthesis of oxindoles **118** from *N*-alkyl,*N*-arylacrylamides **60** and carbazates **117** (Scheme 24) [112–113]. Both protocols utilized a catalytic loading of FeCl_2_·4H_2_O mediated by *tert*-butyl hydroperoxide. The scopes of both reactions were broad and tolerated a variety of functional groups; however, both groups noted unsubstituted terminal alkenes and *N*-arylacrylamides **60** with a free N–H did not undergo this transformation. Interestingly, Du’s protocol tolerated substrates with a free carbinol moiety. On the other hand, Yu reported the same substrate failed to produce any product owing it to oxidative instability, yet more must be at play. Since 2014, modifying the reaction conditions has allowed for several different difunctionalization reactions of alkenes through the denitrogenative radical generation of carbazates. The subsequent radical has been shown to undergo coupling with oxygen sources like peroxides [114–115] and air [116].

**Scheme 25:**
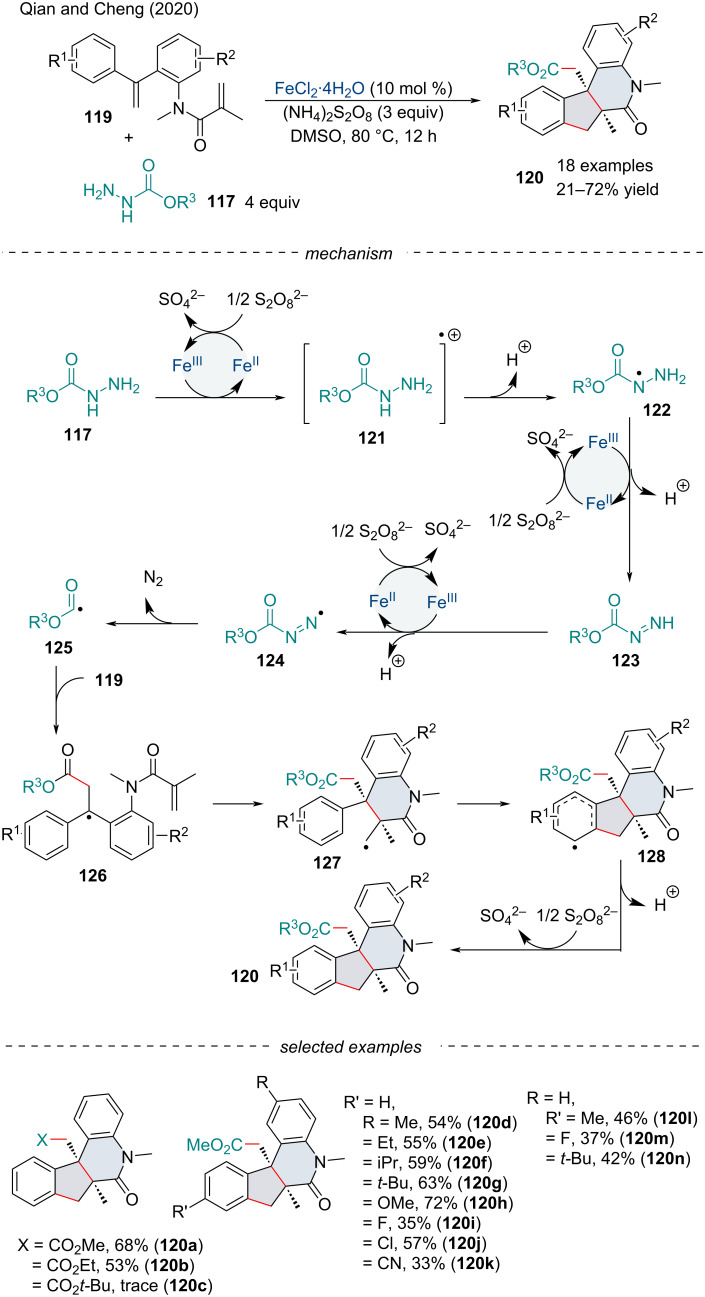
Iron-catalyzed radical 6-*endo* cyclization of dienes **119** with carbazates **117**.

In 2020, Qian and Cheng investigated the cascade cyclization of dienes **119** with alkyl carbazates **117** for the synthesis of fused nitrogen heterocyclic compounds **120** (Scheme 25) [117]. Diene substrates possessing EDGs reacted smoothly under the optimized conditions while their electron-deficient counterparts delivered the products in diminished yields. This transformation was sensitive to steric hindrance, as *ortho*-substituted aryl species and bulky alkyl carbazates failed to react under these reaction conditions. Based on control experiments, the authors proposed a tentative catalytic cycle. Initially, in the presence of an Fe(II) species and S_2_O_8_^2−^, a cascade of SET reactions between the alkylcarbazate and the Fe catalyst will lead to the formation of the alkoxycarbonyl **125**. Regioselective addition of the radical across the electron-neutral olefin will generate the radical intermediate **126**, followed by the 6-*endo* radical cyclization with the activated alkene **127**. Ring closing with the *ortho*-carbon of the aryl ring generates aryl radical **128** which was confirmed not to be the rate-determining step by kinetic isotope effect studies. Subsequently, **128** is oxidized by S_2_O_8_^2−^ and deprotonated to form the desired product **120**.

#### Homolytic-cleavage-initiated C–C/C–C coupling

**Scheme 26:**
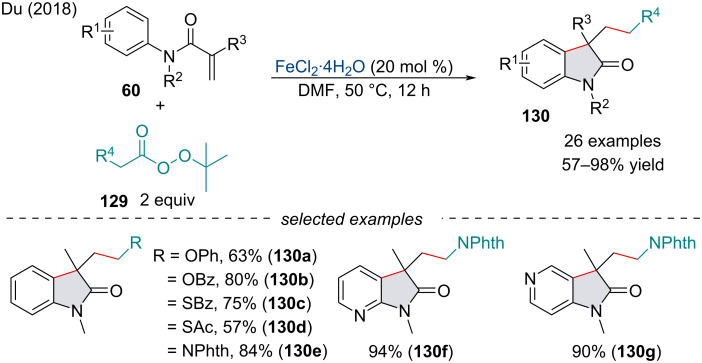
Iron-catalyzed decarboxylative synthesis of functionalized oxindoles **130** with *tert*-butyl peresters **129**.

In 2018, Du and co-workers explored the decarboxylative radical addition/cyclization of *tert*-butyl peresters **129** and *N*-arylacrylamides **60** for the synthesis of oxindoles **130** (Scheme 26) [118]. The scope of the reaction was broad and tolerated a variety of functional groups with neither EDGs nor EWGs altering the reactivity of the acrylamide. Once synthesized, the authors demonstrated the oxindoles could be transformed into fused indoline–heterocycle frameworks in good yield, an attractive scaffold found in many biologically active compounds [119].

**Scheme 27:**
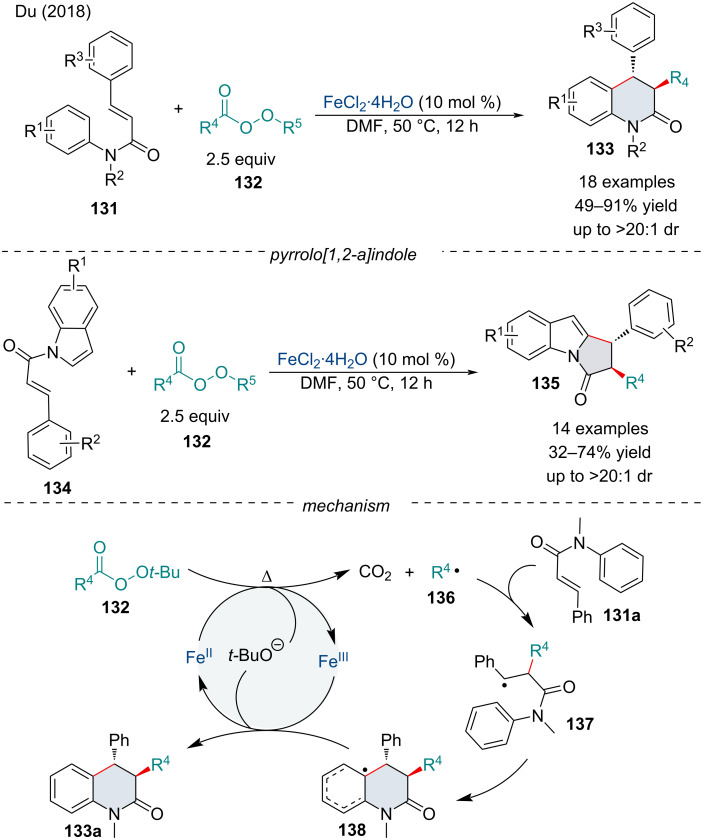
Iron‑catalyzed decarboxylative alkylation/cyclization of cinnamamides **131**/**134**.

In the same year, the Du group investigated the FeCl_2_·4H_2_O decarboxylative radical alkylative cyclization of cinnamamides **131**/**134** as an expedient approach towards dihydroquinolinone **133** and pyrrolo[1,2-*a*]indole **135** analogues in good yield and excellent diastereoselectivity (Scheme 27) [120]. In terms of the dihydroquinolinones **133**, acrylamide starting materials **131** containing EWGs or EDGs all proceeded well, producing the cyclized products with excellent diastereoselectivity. Likewise, the scope of peresters investigated was broad and well received; however, a lower reactivity was observed for sterically demanding alkyl groups. Overall, when the protocol was applied towards the synthesis of pyrrolo[1,2-*a*]indoles **135**, product yields were slightly diminished; however, the scope was equally as broad and tolerated most functional groups. Sterically demanding peresters were shown to react poorly with the indole starting material **134**, with tertiary peresters failing to react. The mechanism begins with an outer-sphere SET from Fe(II) to perester **132** leading to the O–O bond cleavage, generating the reactive alkyl radical **136**, Fe(III), CO_2_, and *t*-BuO^−^. Addition of **136** across the alkene **131a** generates radical intermediate **137**. Subsequently, intramolecular cyclization of **137** generates radical intermediate **138** which then successively undergoes a SET of Fe(III) and deprotonation by *t*-BuO^−^ to give the annulated product **133a** and regenerates the Fe(II) active catalyst.

**Scheme 28:**
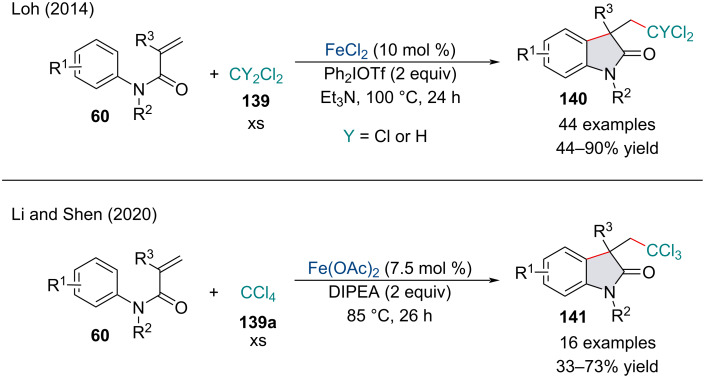
Iron-catalyzed carbochloromethylation of activated alkenes **60**.

In 2014, the Loh group reported an FeCl_2_-catalyzed carbochloromethylation of activated alkenes **60** (Scheme 28) [121]. The reaction was amenable to a range of commercially available chlorinated methane units **139**; however, CH_2_Cl_2_ and CCl_4_ (**139a**) performed the best and delivered the oxindole products **140** in good to excellent yield. The authors noted the use of the diaryliodonium salt Ph_2_IOTf was critical, with no reaction being observed in its absence. In 2020, Li and Shen reported a similar transformation for the synthesis of chloro-containing oxindoles **141** (Scheme 28) [122]. Interestingly, the authors reported the reaction was able to operate in the absence of any external oxidants under an inert atmosphere. Although not investigated, Loh’s transformation most likely begins with the generation of an aryl radical from the reduction of the diaryliodonium salt with Fe(II) which subsequently abstracts a hydrogen from CH_2_Cl_2_ to generate an alkyl radical. Perchlorinated species **139a**, like the substrates investigated by Li and Shen, most likely undergo thermal homolytic bond cleavage and do not rely on a radical initiator.

**Scheme 29:**
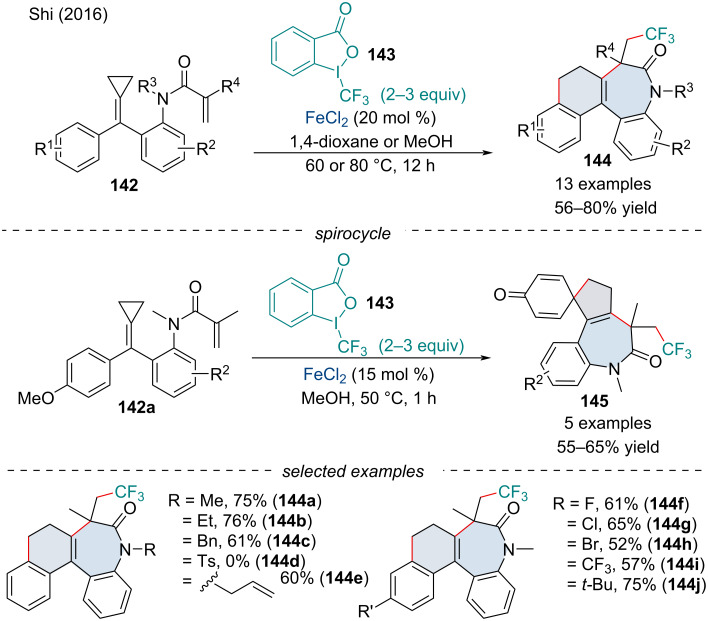
Iron-catalyzed trifluoromethylation of dienes **142**.

In 2016, Shi and co-workers investigated the trifluoromethylation cascade of acrylamide-tethered alkylidenecyclopropanes **142** for the synthesis of polycyclic benzazepine derivatives **144** (Scheme 29) [123]. The authors noted electron-withdrawing substituents were detrimental to the efficacy of the reaction with electron-withdrawing *N*-substituents failing to react. When a methoxy group was installed at the *para*-position of the aryl ring, a spirocyclic product **145** was formed via a radical cyclization/dearomatization process. Mechanistic investigations revealed the reaction operates through a radical pathway.

**Scheme 30:**
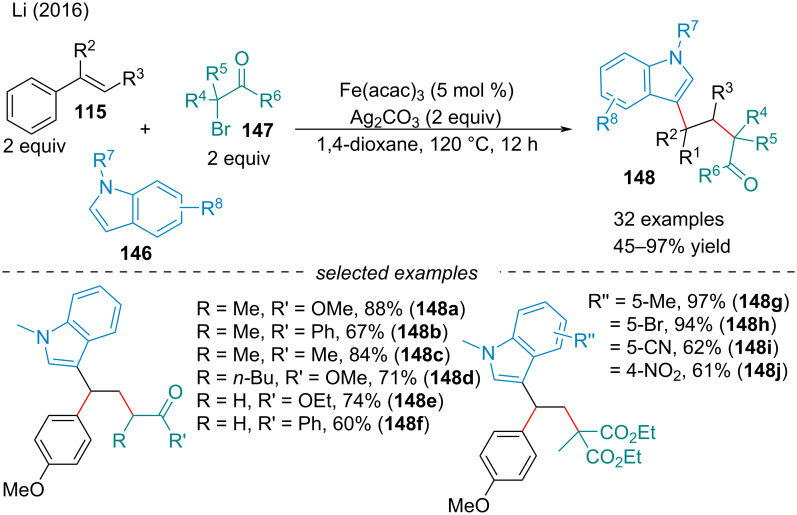
Iron-catalyzed, silver-mediated arylalkylation of conjugated alkenes **115**.

In 2017, the Li group described a Ag-mediated, Fe-catalyzed alkylarylation of styrene derivatives **115** with α-carbonyl alkyl bromides **147** and indole derivatives **146** (Scheme 30) [124]. Although the reaction operated in the absence of the iron catalyst, its use is crucial for high yielding reactions. Preliminary mechanistic studies suggest the reaction proceeds through a radical addition of the carbon-centered alkyl radical across the alkene to afford the benzylic radical. Oxidation of the corresponding radical affords the benzylic carbocation which is attacked by the indole nucleophile. The authors applied the reaction methodology to pyrrole as a substrate; however, only one example was given in a 50% yield. Further examining of other potential arenes capable of undergoing electrophilic aromatic substitution would expand the applicability of the reaction.

#### Carboazidation

**Scheme 31:**
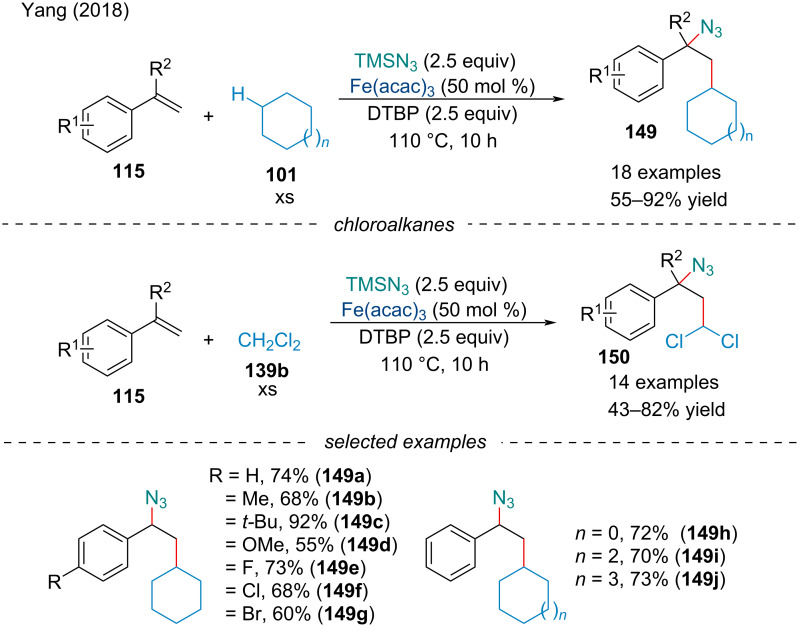
Iron-catalyzed three-component carboazidation of conjugated alkenes **115** with alkanes **101**/**139b** and trimethylsilyl azide.

In 2018, Yang investigated the three-component carboazidation of styrene derivatives **115** with alkanes **101**/**139b** and trimethylsilyl azide for the synthesis of chain extended azides **149** and γ-azido chloroalkanes **150** in good yield (Scheme 31) [125]. The electronic nature of the alkene had no clear effect on the reactivity of the system; however, no product was detected when unactivated alkenes like when cyclohexene was used. This strategy was also explored using chloroalkanes to form di- and trichlorinated products **150**. Despite previous reports demonstrating dichloromethane **139b** in the presence of peroxide [126] and iron salts [127] form 1,1,1- and 1,1,1,3-substituted chloroalkanes, under the authors’ optimized reaction conditions only the carboazidation product was observed. In the same year, the Xu laboratory demonstrated Togni’s reagent could be employed for the synthesis of γ-azido fluoroalkanes [128].

In 2019, Chu and co-workers demonstrated the three-component carboazidation of alkenes with chloroalkanes and trimethylsilyl azide could be solvent-tuned [129]. In neat CH_2_Cl_2_, the reaction produced the expected β-trichloromethyl alkyl azide; however, the reaction was chemoselective for diazidation when *tert*-butanol was used as co-solvent. The authors hypothesized the presence of the alcohol suppresses the polar-unmatched HAT process from forming CHCl_2_ radicals [130].

**Scheme 32:**
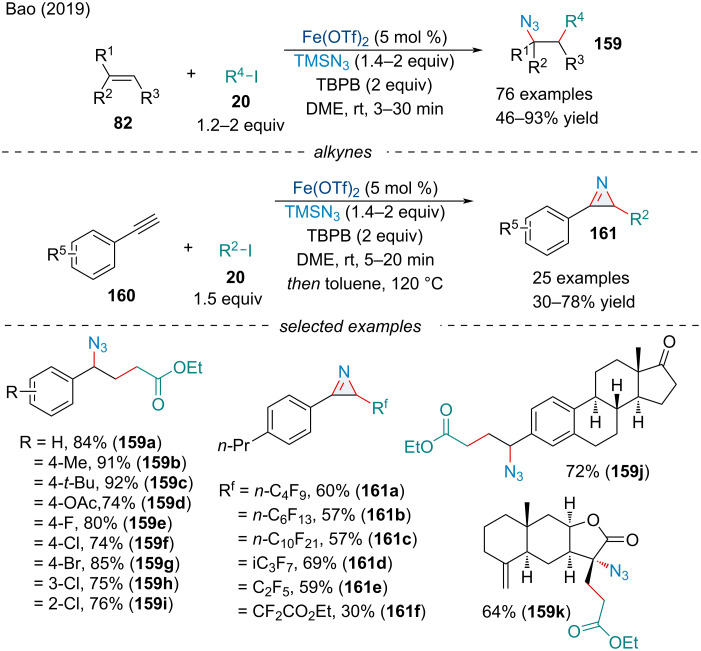
Iron-catalyzed carboazidation of alkenes **82** and alkynes **160** with iodoalkanes **20** and trimethylsilyl azide.

In 2019, the Bao group demonstrated alkyl iodides **20** were suitable radical precursors for the carboazidation reaction (Scheme 32) [131]. Additionally, the authors demonstrated the carboazidation of alkynes **160**, a challenging reaction which has only had success under copper catalysis [132]. Electron-rich alkyl iodides did not produce the desired product with only perfluorinated and ester-containing alkyl iodides **20** working well. Despite the limited applicability of nucleophiles, the reaction was extremely fast, typically finishing in under 10 minutes. To show the synthetic utility of their reaction, the authors studied the one-pot conversion of the vinyl azides to 2*H*-azirines **161**. The carboazidation reaction for the aryl alkynes was completed in a comparable amount of time. A myriad of different functionalized π-systems was tolerated by the reaction, demonstrating its applicability in late-stage functionalization. The Bao group has since demonstrated many other carbon-centered radicals were amenable in the carboazidation reaction of alkenes including diacylperoxides [133] and aldehydes [134].

**Scheme 33:**
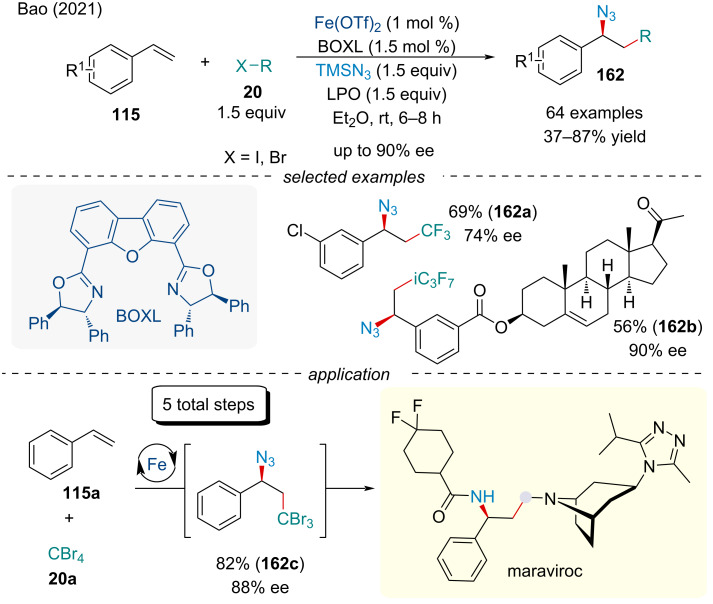
Iron-catalyzed asymmetric carboazidation of styrene derivatives **115**.

In 2021, the Bao group followed up on their previous work and developed an asymmetric carboazidation of styrene derivatives **115** (Scheme 33) [135]. The authors propose the enantioselectivity originates from the diastereoisomeric azido group transfer from the Fe(III) center to the benzylic radical. Not only did the described methodology produce enantiopure products in up to 90% ee, the reactivity and applicability outperformed the racemic variant. Application of this methodology was applied towards the total synthesis of maraviroc, an anti-HIV drug, which was synthesized in 5 steps starting from styrene (**115a**) and CBr_4_ (**20a**).

#### Carboamination

**Scheme 34:**
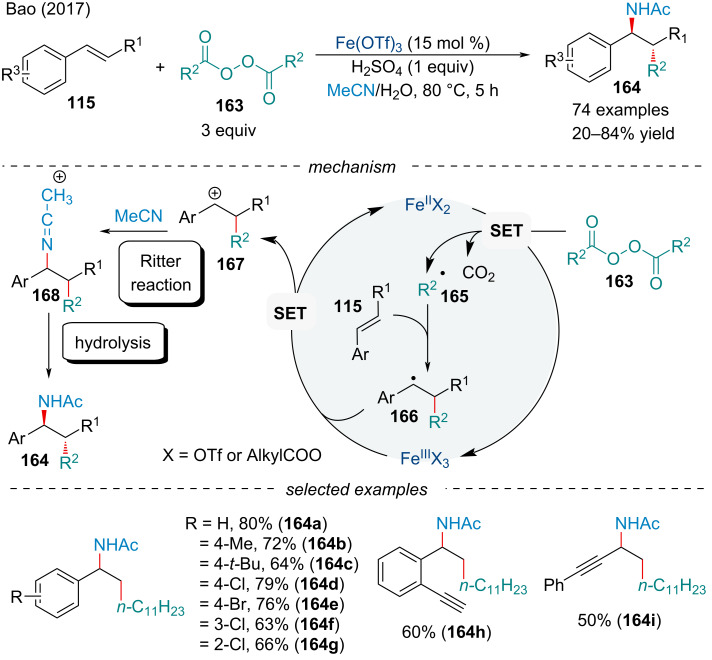
Iron-catalyzed carboamination of conjugated alkenes **115** with alkyl diacyl peroxides **163** and acetonitrile.

In 2017, the Bao group investigated the carboamination of activated alkenes **115** with alkyl diacyl peroxides **163** and acetonitrile (Scheme 34) [136]. Their efficient protocol featured a broad substrate scope, including diversely functionalized styrene derivatives, various alkyl diacyl peroxides, and a few different nitrile solvents which provided the desired carboamination product **164**. By using methyl cinnamate derivatives, the reactions were highly diastereoselective for the formation of 1,2-*anti* carboamination products. Notably, the Ritter reaction was found to be the origin of diastereoselectivity on the basis of a density functional theory (DFT) mechanistic study. The authors proposed a radical-polar crossover mechanism. First, a SET from Fe(II) to the alkyl diacyl peroxide generates the alkyl acyloxy radical which decarboxylates to afford the alkyl radical **165**. Addition of the radical across the alkene affords the benzylic radical **166** which is oxidized by Fe(III) to a carbocation species **167**. Subsequent attack by the nitrile affords the nitrilium ion **168** which upon hydrolysis gives the final product **164**.

Oxime esters and ethers represent a widely used starting material for the generation of nitrogen-containing heterocycles. Due to the breadth of reactions viable from iminyl radicals, generating through the homolytic cleavage of the N–O bond, a number of Fe-catalyzed domino reactions have been investigated.

**Scheme 35:**
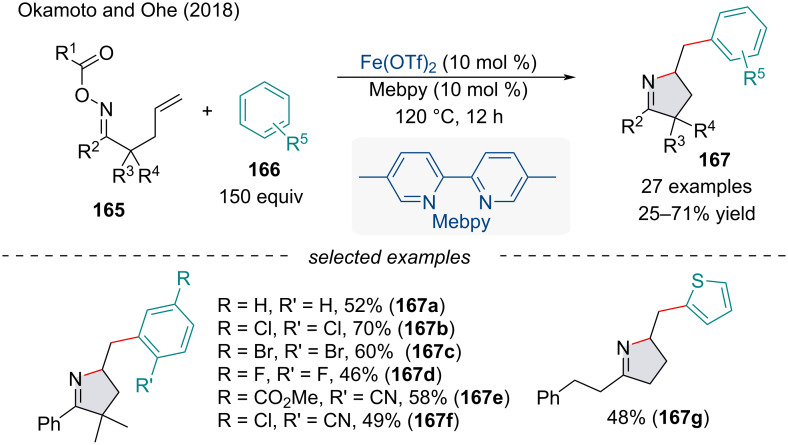
Iron-catalyzed carboamination using oxime esters **165** and arenes **166**.

In 2018, Okamoto and Ohe reported an iminoarylation of γ,δ-unsaturated oxime esters **165** with arenes **166** (Scheme 35) [137]. The reaction most likely involves an iminyl radical which undergoes a 5-*exo*-*trig* cyclization with the alkene to form the alkyl radical intermediate. Homolytic aromatic substitution (HAS) with the arene will afford the final functionalized product. Interestingly, electron-poor, electron-rich, and heteroarenes were all well-tolerated; however, monosubstituted aryl rings suffered from low regioselectivity which is in accordance with HAS-type reactions [138].

In 2020, the Wu group expanded on the radical cyclization chemistry of γ,δ-unsaturated oxime esters, describing a carbonylative cyclization with amine nucleophiles [139]. Following an iminyl radical-mediated intramolecular 1,5-cyclization, the subsequent alkyl radical could propagate across carbon monoxide, eventually terminating with an alkyl or aryl amine. In the following year, the Li group described an interesting radical cyclization of β,γ-unsaturated oximes using iron(III) nitrate at a 50% catalytic loading in which the catalyst also acted as a source of nitrate ions for the reaction [140]. Upon oxidation of the alkyl radical, the Fe(III) species is reduced to Fe(II) releasing a nitrate anion which attacks the now electrophilic carbocation for a net iminyl-nitrooxylation reaction [140].

**Scheme 36:**
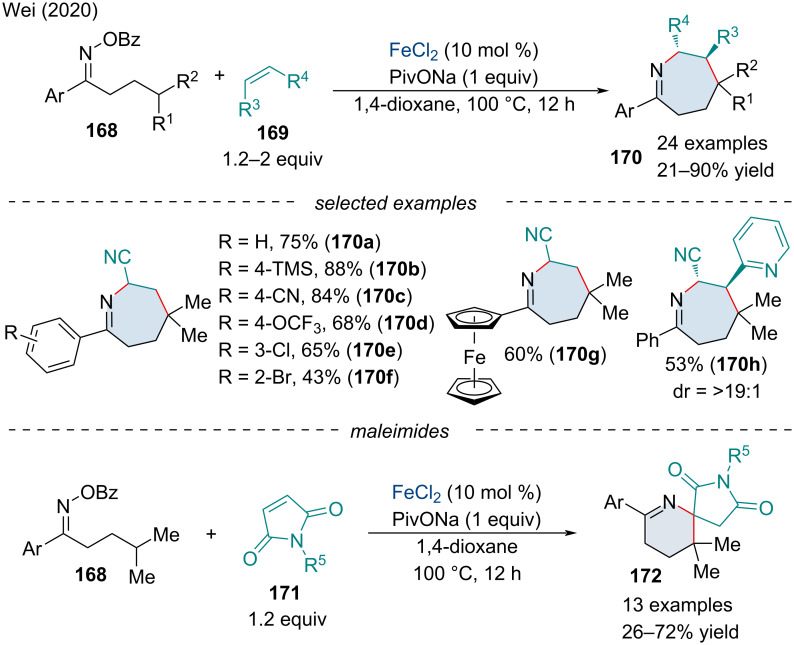
Iron-catalyzed iminyl radical-triggered [5 + 2] and [5 + 1] annulation reactions with oxime esters **168** and alkenes **169**.

In 2020, Wei and co-workers studied an iminyl radical-triggered 1,5-hydrogen atom transfer (HAT) and [5 + 2] annulation processes for the synthesis of azepine derivatives **170** (Scheme 36) [141]. The reaction was tolerable of both electron-donating and electron-withdrawing substituents on the oxime; however, the reaction required highly activated alkenes to proceed. 1,2-Disubstituted alkenes were tolerated and were diastereoselective for the *anti*-addition product. When maleimides **171** were used as the 2-carbon coupling partner, a [5 + 1] annulation was observed generating spiro succinimidetetrahydropyridine derivatives **172**. To understand the chemoselectivity of the reaction, the authors performed a DFT mechanistic study. After the iminyl radical is generated it will undergo a 1,5-HAT to form the more stable alkyl radical which will add across the alkene. In the case of activated alkenes, the species will undergo a 7-*exo-trig* cyclization. On the other hand, it is energetically more favorable for the maleimide species to sequentially undergo a 1,4-HAT, 1,4-HAT, 1,6-HAT and intramolecular cyclization to form the 6-membered ring. Further, by applying different coupling partners, both bi- [142] and tricyclic [143] fused heterocyclic frameworks have been synthesized through iminyl radical cascade cyclization and annulation reactions.

#### Carbooxygenation

**Scheme 37:**
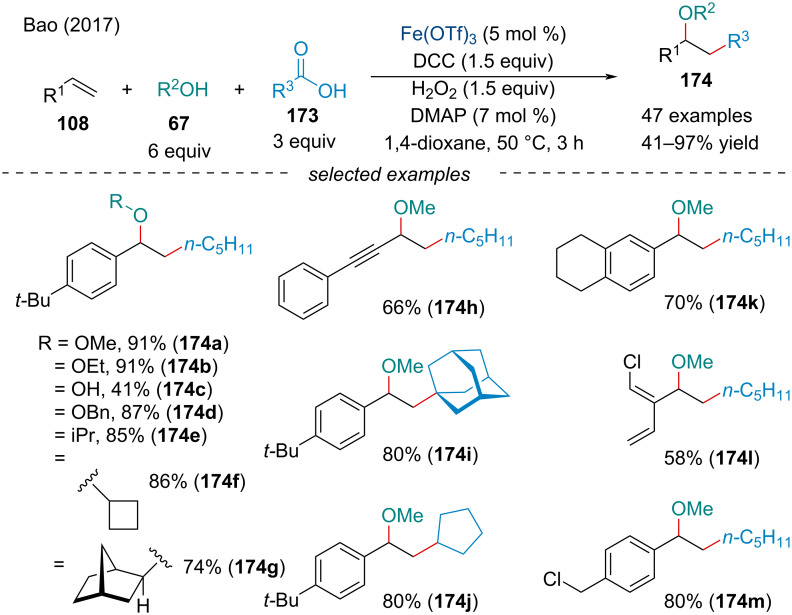
Iron-catalyzed decarboxylative alkyl etherification of alkenes **108** with alcohols **67** and aliphatic acids **173**.

In 2017, the Bao group reported the first decarboxylative alkyl etherification of alkenes **108** with alcohols **67** and aliphatic acids **173** (Scheme 37) [144]. Through a DCC-mediated dehydrogenative condensation with hydroperoxides, carboxylic acids could generate alkyl diacyl peroxides and peresters in situ. Decarboxylation followed by radical addition across the alkene **108** would generate a succeeding alkyl radical. Oxidation of the alkyl radical would form the corresponding carbocation which could be captured by oxygen nucleophiles **67** to afford the carboetherification product.

**Scheme 38:**
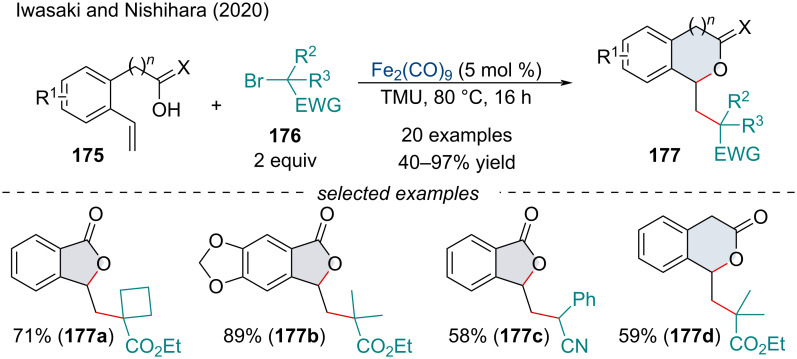
Iron-catalyzed inter-/intramolecular alkylative cyclization of carboxylic acid and alcohol-tethered alkenes **175**.

In 2020, Iwasaki and Nishihara investigated the inter-/intramolecular alkylative cyclization of carboxylic acid and alcohol-tethered alkenes **175** (Scheme 38) [145]. Control experiments determined the reaction involved a radical process. The proposed mechanism comprised a conventional generation of the alkyl radical through a SET which subsequently adds across the alkene. Compared to Guo’s and Li’s seminal reports (Scheme 19), the authors propose the intramolecular cyclization proceeds via the nucleophilic attack of a brominated or cationic benzylic position rather than a radical cyclization. In 2021, Tang and Zhang demonstrated a similar radical annulation of unsaturated carboxylic acids with disulfides for the synthesis of γ-lactones [146].

**Scheme 39:**
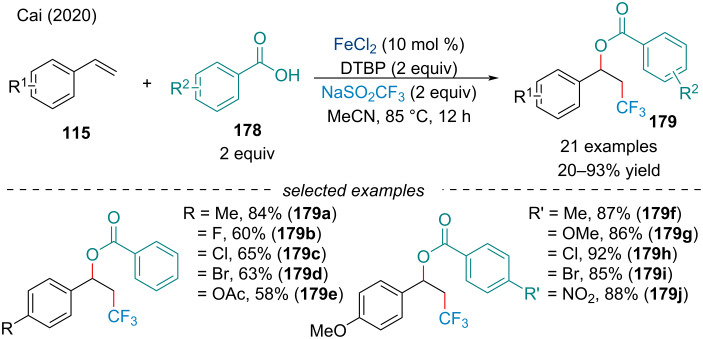
Iron-catalyzed intermolecular trifluoromethyl-acyloxylation of styrene derivatives **115**.

In 2020, Cai and co-workers reported a three-component intermolecular trifluoromethyl-esterification of activated alkenes **115** with NaOTf and aryl carboxylic acids **178** (Scheme 39) [147]. Notably, the use of NaOTf as a CF_3_ source, compared to pre-prepared trifluoromethylating agents like Togni’s reagent, is ideal because of its stability, low toxicity, and cost [148]. In terms of scope, the electronic nature of the benzoic acid had no effect on the reaction. On the other hand, only electron-rich styrene derivatives were found to work well in the reaction, with unactivated and aliphatic alkenes having little to no reactivity.

#### Carbohalogenation

**Scheme 40:**
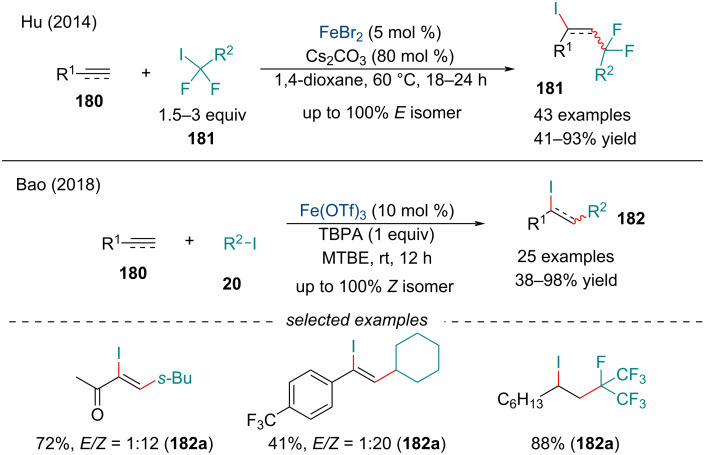
Iron-catalyzed carboiodination of terminal alkenes and alkynes **180**.

In 2009, Liu’s group established benzyl halides can be added across aryl alkynes for the synthesis of alkenyl halides [149]. Similarly, the addition of perfluoroiodides **181** across π-systems **180** was reported by Hu in 2014 (Scheme 40) [150]. Carboiodination of terminal alkynes was stereoselective for the formation of the *E*-isomer **181**. Compared the Liu’s report, this reaction was applicable to both aliphatic alkenes and alkynes. To show the applicability of this transformation, the authors performed a series of cross-coupling reactions, demonstrating the neighboring perfluoroalkyl groups did not impede the reaction. In 2018, Bao and co-workers expanded on the carboiodination reaction (Scheme 40) [151]. Their method allowed for the general alkyl chains, as well as ester and cyano moieties, to be added across the alkene. Both aryl and aliphatic alkenes and alkynes **180** were found to work well in the transformation. Interestingly, alkyl iodides **20** provided the corresponding vinyl iodide with overall *anti* addition. When the iodo-substrate was equipped with an electron-withdrawing group, up to 100% of the *syn* product **182** was observed.

**Scheme 41:**
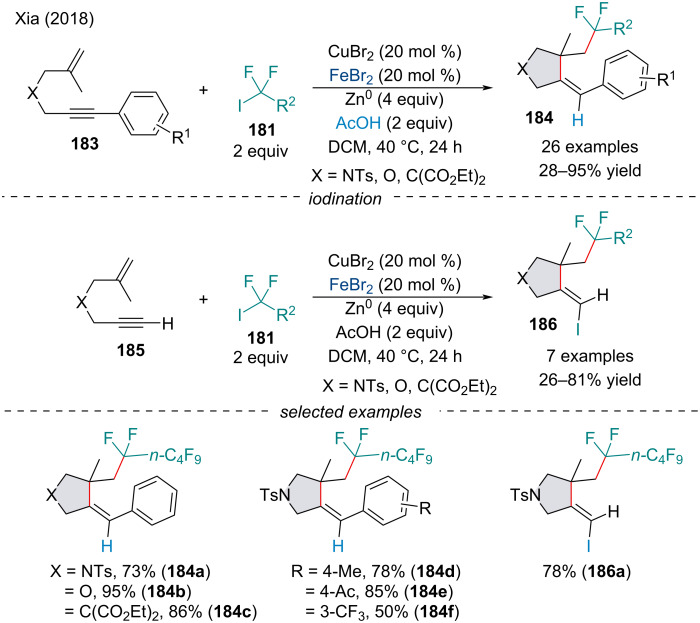
Copper/iron-cocatalyzed cascade perfluoroalkylation/cyclization of 1,6-enynes **183**/**185**.

In 2018, the Xia laboratory reported a Cu/Fe-cocatalyzed cascade perfluoroalkylation/cyclization of 1,6-enynes **183**/**185** for the synthesis of fluoroalkylated pyrrolidines and benzofurans **184**/**186** (Scheme 41) [152]. When either catalyst was used on its own, the reaction had little to no success, demonstrating the necessity for tandem catalysis. When terminal alkynes **185** were used, the transformation was terminated by an iodine transfer process rather than hydrogen abstraction to furnish vinyl iodides **186**. When tetrasubstituted vinyl iodides were synthesized and exposed to the reaction conditions, a deiodination occurred, suggesting the vinyl iodide may be an intermediate in the mechanism. The authors propose sequential addition of the alkyl radical across the alkene followed by the alkyne produces the cyclization intermediate. This intermediate may undergo halogen abstraction to form the vinylic iodide. If an internal alkyne is used, the iodide may undergo oxidative addition followed by reduction and protonation. Alternatively, the internal alkyne-cyclized intermediate may simply proceed through a vinylic metallic species which is reduced by zinc metal and protonated.

#### Carbosilylation

**Scheme 42:**
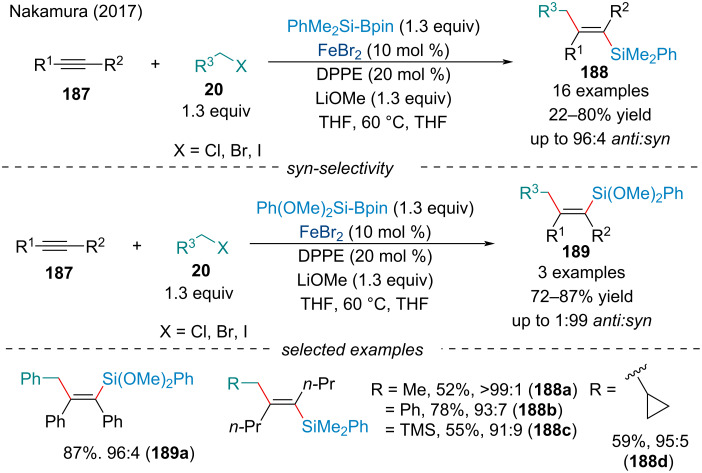
Iron-catalyzed stereoselective carbosilylation of internal alkynes **187**.

In 2017, the Nakamura group demonstrated the carbosilylation of alkynes **187** (Scheme 42) [153]. The protocol allowed for the use of various alkyl halides as electrophiles. Interestingly, both *syn-* and *anti*-selective carbosilylation could be achieved by altering the reaction conditions and employing different silylboranes. The authors hypothesized the oxygen functionality on the silylborane coordinates with the iron center to form a more stable, chelated *Z*-alkenyliron species. Trialkylsilanes were incompatible with the reaction, suggesting a Lewis-acidic silyl center is preferred in the reaction pathway; hence, the necessity for at least one phenyl substituent.

#### Carbosulfinylation

**Scheme 43:**
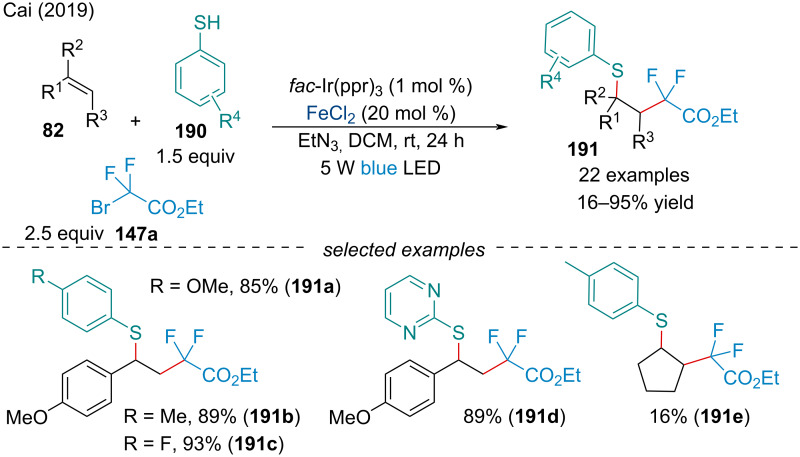
Synergistic photoredox/iron catalyzed difluoroalkylation–thiolation of alkenes **82**.

In 2019, the Cai laboratory demonstrated the first difluoroalkylative–thiolation of alkenes **82** by Fe-facilitated visible-light photoredox catalysis (Scheme 43) [154]. During optimization, the authors noted both the photocatalyst and iron salt critically affected the efficacy of the reaction. When no photocatalyst was present the reaction failed entirely, and only a trace amount of desired product was obtained in the absence of the iron salt. Consistent with many Fe-catalyzed difunctionalization reactions, only activated alkenes were tolerated in the reaction, with aliphatic olefins having reduced reactivity. Both electron-rich and deficient thiophenols **190** were shown to react smoothly under the reaction conditions; however, future work should be done examining the applicability of the reaction with aliphatic thiol coupling partners. In the same year, Cai and co-workers elaborated on their previous study to explore the potential of amines as coupling partners in the three-component difluoroalkylamination of alkenes mediated by photoredox and iron cooperative catalysis [155]. Similar reactivity trends were observed for the alkene component of the reaction; however, electron-deficient arylamines were found to be unreactive.

#### Hetero-difunctionalization

In recent years, many advances in two and three-component hetero-difunctionalization reactions have been made, offering a powerful method for increasing molecular complexity in organic synthesis. Recent accounts have demonstrated Fe-catalyzed cascade reactions can promote the incorporation of several heteroatoms across π-systems, quickly assembling multiple carbon–heteroatom bonds in a single reaction.

#### Aminoazidation

**Scheme 44:**
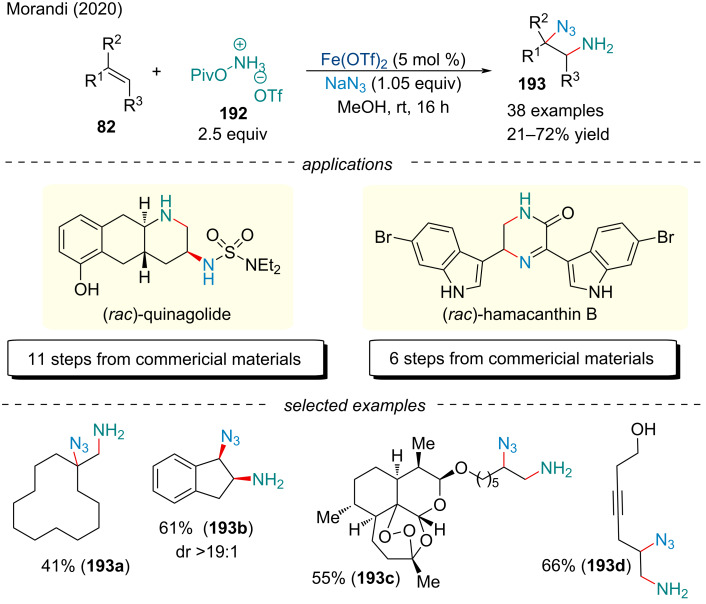
Iron-catalyzed three-component aminoazidation of alkenes **82**.

The synthesis of undifferentiated vicinal diamines and diazides has been described by Xu and co-workers as powerful tool for accessing symmetrical difunctionalized compounds; these reactions suffer where two chemically distinct amino groups need to be orthogonally synthesized [156]. In 2020, the Morandi group developed an Fe(OTf)_2_-catalyzed aminoazidation of alkenes **82** for the synthesis of unprotected primary 2-azidoamines **193** (Scheme 44) [157]. The catalytic process demonstrated a considerable scope, with good yields, and with cyclic alkenes, good diastereoselectivity. Further, the mild conditions allowed for superior functional group tolerance. Its potential for early- and late-stage functionalization is highlighted with the artemether derivative **193c** where the highly oxidized cage structure and the sensitive peroxo group were left intact. Application of this methodology to the total synthesis of (±)-hamacanthin B and (±)-quinagolide further demonstrated the broad synthetic potential.

Efforts to expand the scope of aminoazidation reactions were met with success in 2021, when a report from the Bao group described a method for accessing chiral organo azides [158]. Using a chiral BOX ligand, the authors demonstrated a large scope with excellent yields (up to 98%) and enantioselectivity (up to 96% ee). Unlike Morandi’s report (Scheme 44) [157], the authors only included styrene derivatives in the scope of potential alkenes. Adaptation of the reaction conditions also allowed for the synthesis of enantioenriched diazido products [158].

**Scheme 45:**
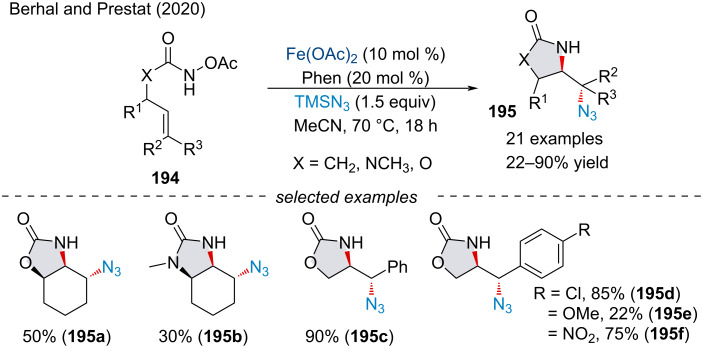
Iron-catalyzed intra-/intermolecular aminoazidation of alkenes **194**.

In 2020, Berhal and Prestate demonstrated a tandem intra/intermolecular aminoazidation of unactivated alkenes **194** for the synthesis of a variety of heterocyclic scaffolds **195** (Scheme 45) [159]. Although yields of up to 90% were achieved, yields were typically moderated at best. Despite the low yield, the protocol offered a more economically and ecologically sustainable method for the production of azido-containing imidazolidinone, oxazolidinone, and pyrrolidinones which avoided highly shock and friction sensitive reagents.

#### Oxyazidation

**Scheme 46:**
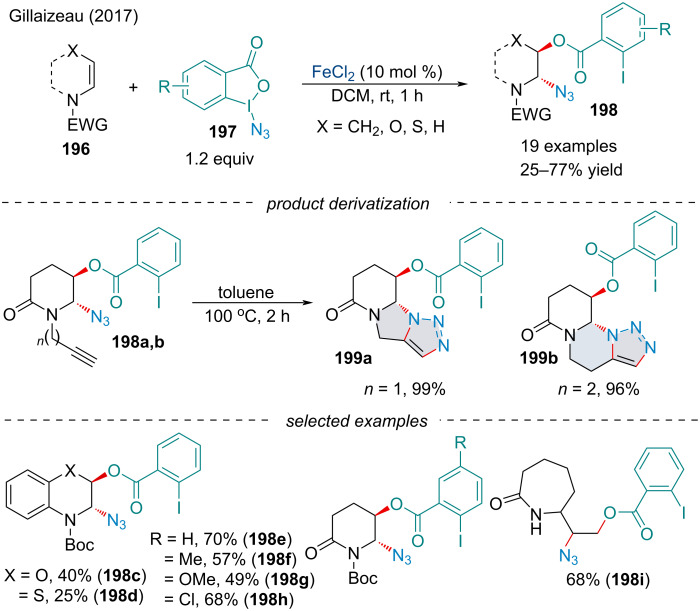
Stereoselective iron-catalyzed oxyazidation of enamides **196** using hypervalent iodine reagents **197**.

In 2017, Gillaizeau and co-workers investigated the intermolecular oxyazidation of enamides **196** using azidobenziodoxolone derivatives **197** (Scheme 46) [160]. Both cyclic and acyclic enamides were amenable to the reaction and were compatible with a variety of functional groups. Interestingly, the reaction was mild enough to afford difunctionalized products in the presence of alkynes **198a**,**b** which could be converted into tricyclic triazine derivatives **199a/199b** via a thermal Huisgen 1,3-dipolar cycloaddition in quantitative yield.

#### Aminooxygenation

**Scheme 47:**
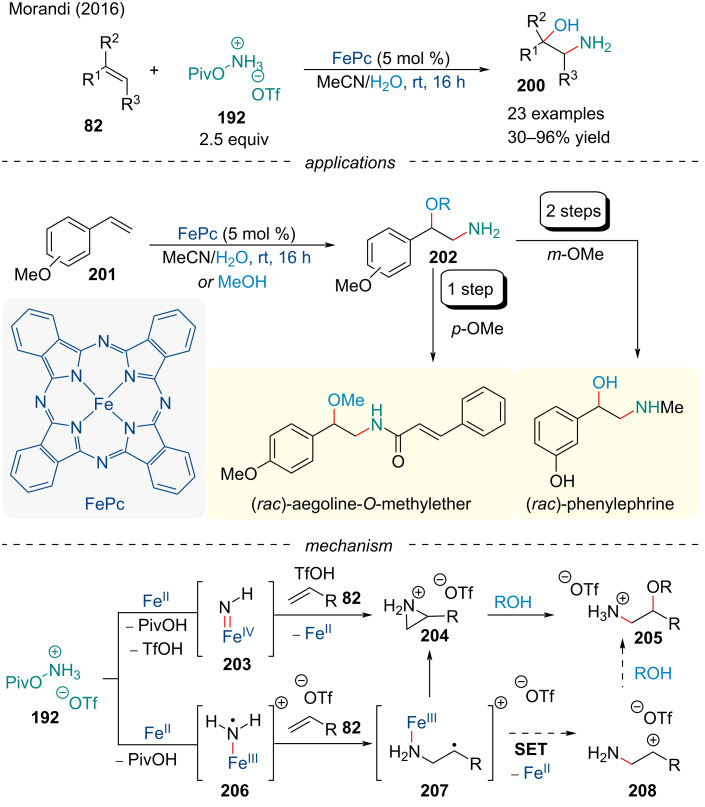
Iron-catalyzed aminooxygenation for the synthesis of unprotected amino alcohols **200**.

In 2016, the Morandi group reported the aminohydroxylation of alkenes **82** for the synthesis of unprotected amino alcohols **200** in good yield (Scheme 47) [161]. Their account relied on the use of PivONH_3_OTf **192** as an easily accessible aminating reagent. Various functionalized styrene derivatives afforded the corresponding product in good yield; however, strongly electron-withdrawing groups were detrimental to the reaction. Further, under the optimized reaction conditions, most unactivated alkenes had little to no reactivity. The method could be extended to other oxygen nucleophiles for access to amino ethers; however, the scope of applicable alcohols was low. Application of this protocol allowed for the rapid synthesis of aegoline-*O*-methyl ether and phenylephrine in good yield. Through a series of mechanistic studies, the authors propose two potential pathways. First, the iron catalyst will react with the hydroxylamine salt to form either an Fe(IV) nitrene complex **203** or an Fe(III) aminyl species **206**. The Fe(IV) nitrene can add across the alkene **82** to form a protonated aziridine **204** which is opened by intermolecular nucleophilic attack by the oxygen nucleophile. Alternatively, the Fe(III) aminyl species adds across the alkene **82** to form the radical intermediate **207**. Oxidization by a SET forms the carbocation **208** which is captured by the oxygen nucleophile.

Efforts to expand the scope of aminohydroxylation reactions were met with success in 2019, when a report from the Arnold group described a method for accessing enantioenriched amino alcohols [162]. Using an engineered hemoprotein biocatalyst based on a thermostable cytochrome c. Not only could the engineered protein deliver the product in good yield and excellent enantioselectivity (up to 90% ee), but the protocol also boasts turnover numbers up to 2500. Similar to Morandi’s account [161], the reaction’s efficacy drops significantly when electron-deficient or unactivated alkenes were examined.

#### Aminohalogenation

**Scheme 48:**
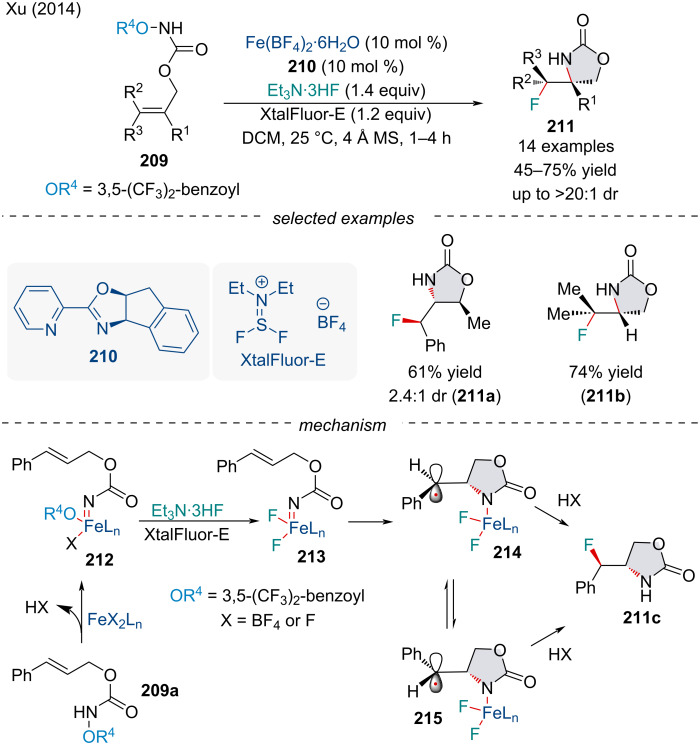
Iron-catalyzed intramolecular aminofluorination of alkenes **209**.

In 2014, the Xu group investigated a diastereoselective Fe-catalyzed aminofluorination of alkenes **209** to produce cyclic carbamates **211** (Scheme 48) [163]. During optimization, both aminofluorinated and aminohydroxylated [164–165] products were observed; however, the use of carboxylate trapping reagent XtalFluor-E^®^ suppressed the competing aminohydroxylation process. Noteworthy, both *E* and *Z* isomers of the olefin starting material **209** led to the same major diastereomer. Based on the observed stereoconvergence, the reaction likely occurs in a stepwise fashion. Based on a mechanistic investigation, the authors propose a working mechanism. First, the Fe complex will reductively cleave the N–O bond to generate Fe-nitrenoid complex **212**. In the presence of a fluoride source, an anion metathesis converts the nitrenoids to difluoride **213**. Subsequent stepwise cycloamination generates carbon-centered radical intermediate **214** which is in fast equilibrium with **215**. Rotation of the σ-bond at this step could potentially explain the observed stereoconvergence.

**Scheme 49:**
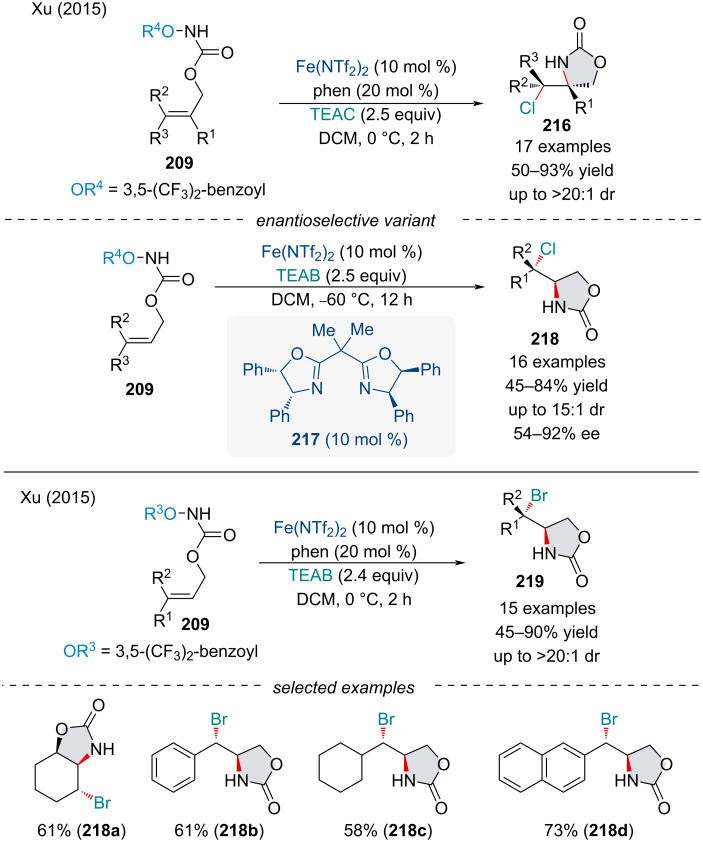
Iron-catalyzed intramolecular aminochlorination and aminobromination of alkenes **209**.

In the following year, the same group explored the diastereoselective intramolecular Fe-catalyzed aminochlorination of alkenes **209** (Scheme 49) [166]. Initially, the authors developed a racemic reaction employing Fe(NTf)_2_ and phenanthroline with tetrabutylammonium chloride (TEAC). In contrast to the work of Bach [167], the corresponding acyl azide failed to react under the reactions conditions and was fully recovered. Expanding the single enantioselective aminofluorination reaction reported in 2014 [163], Xu and co-workers developed an asymmetric Fe-catalyzed aminochlorination of alkenes **209** through the use of a bisoxazole ligand [166]. The reaction regioselectively led to a range of chiral products in moderate to good yields (45–84%) combined with moderate to high enantioselectivities (54–92% ee). In the same year, the authors applied the enantiomeric catalyst to promote the asymmetric intramolecular aminobromination of alkenes **209** with tetraethylammonium bromide (TEAB) [168].

**Scheme 50:**
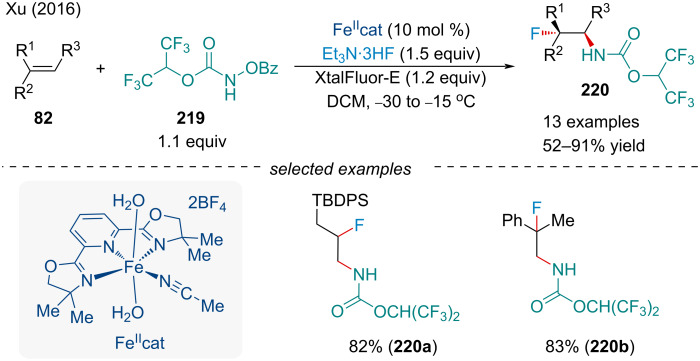
Iron-catalyzed intermolecular aminofluorination of alkenes **82**.

In 2016, the Xu group continued to investigate Fe-catalyzed aminohalogenation reactions of alkenes **82** (Scheme 50) [169]. Using Et_3_NH·3HF as the fluoride source, the authors reported the first intermolecular aminofluorination of alkenes. Similar to their 2014 report [163], the use of XtalFluor-E^®^ as a carboxylate trapping agent was key to the success of the reaction. Various unfunctionalized alkenes, were amenable to the reaction allowing for the production of a wide range of vicinal fluorocarbamates **220** in high regioselectivity [169]. Later, the authors developed an enantioselective Fe-catalyzed intermolecular aminofluorination reaction of indene [169]. A series of acyloxyl carbamates and chiral PyBOX ligands were screened with the *anti*-2-amino fluoride being delivered in poor to good ee. Compared to the racemic variant, the yield and diastereoselectivity of the reaction was slightly diminished.

**Scheme 51:**
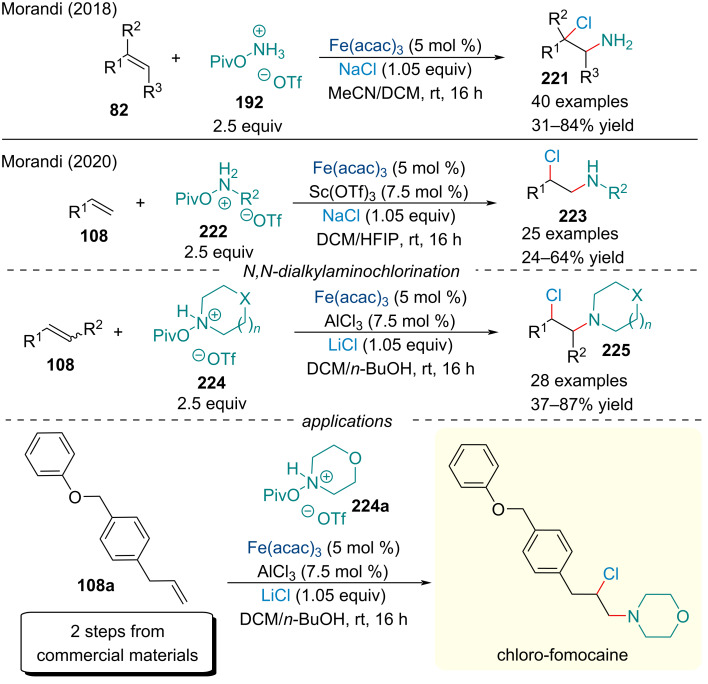
Iron-catalyzed aminochlorination of alkenes **82**.

In 2018, the Morandi group continued studying the Fe-catalyzed amination reactions. The group reported the addition of NaCl enabled the formation of 2-chloroalkylamine products **221** in good yields and excellent *anti*-Markovnikov selectivity (Scheme 51) [170]. In 2020, the scope of this aminochlorination reaction was expanded to include both primary and secondary amines **222**/**224** [171]. Impressively, both methods proceeded under exceedingly mild conditions with excellent functional group tolerance using stable and inexpensive aminochlorinating reagents, applicable for late-stage functionalization. In contrast to their previous report, the reactions displayed broad reactivity and worked well for unactivated internal alkenes. To illustrate the versatility of this protocol, the authors synthesized a chlorinated fomocaine analog in three steps.

#### Phosphinoylazidation

**Scheme 52:**
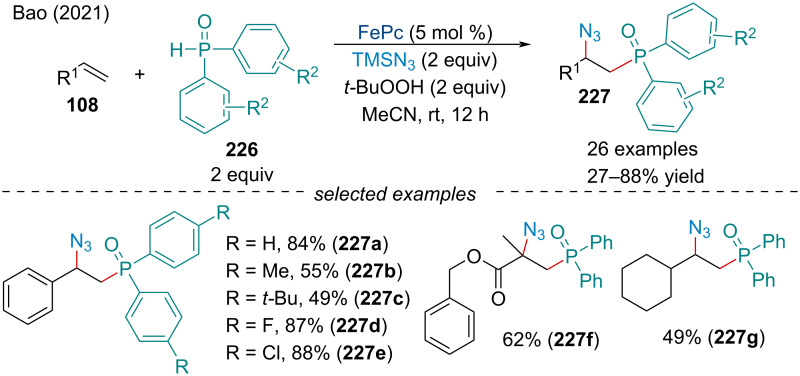
Iron-catalyzed phosphinoylazidation of alkenes **108**.

In 2021, the Bao group described the phosphinoylazidation of alkenes **108** under mild conditions with diarylphosphine oxides **226** and TMSN_3_ (Scheme 52) [172]. Through a DFT investigation, the authors noted the azido transfer from PcFe(III)N_3_ to a benzylic cation has an activation energy of 4.8 kcal/mol. This barrier is remarkably low compared to similar reactions using other earth abundant metals like copper [173] and manganese [174] which have barriers ranging 7.0–10.7 kcal/mol. In terms of scope, only diarylphosphine oxide derivatives were found to be applicable, with dialkylphosphine oxide and phosphonate compounds failing to produce the desired product. Unactivated alkenes were tolerated but less fruitful, most likely due to the instability of the carbocationic intermediate.

#### Aminoselenation

**Scheme 53:**
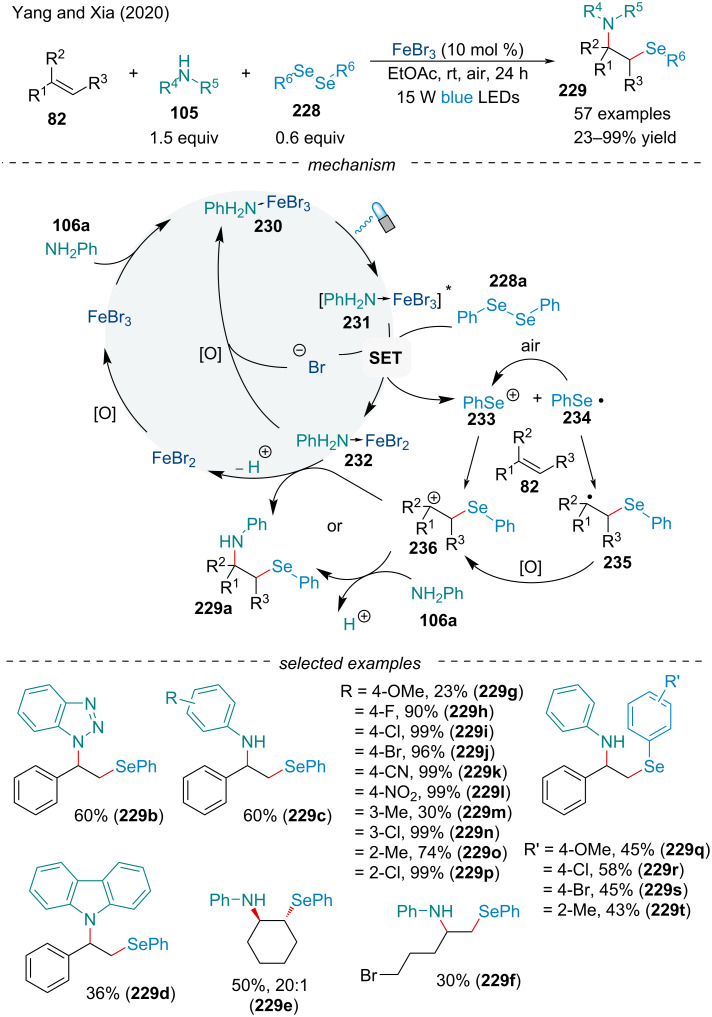
Synergistic photoredox/iron-catalyzed three-component aminoselenation of trisubstituted alkenes **82**.

In 2020, Yang and Xia explored the visible-light enabled, FeBr_3_-catalyzed aminoselenation of alkenes **82** with amines **105** and diselenides **228** under an air atmosphere (Scheme 53) [175]. Excellent functional group tolerance was observed while scoping the reaction out. Primary anilines bearing electron-withdrawing groups typically provided the difunctionalized products in a greater yield compared to electron-rich or secondary anilines. Due to the lack of photo-absorption, aliphatic amines failed to react. Aliphatic diselenides worked, but showed low reactivity compared to aromatic diselenides. Based on mechanistic observations, the initial step is presumed to be the interaction of the amine with the Fe(III) to generate [FeBr_3_·NH_2_Ph] **230** which can be photo-excited to reach the excited state **231**. The latter reacts through a SET oxidation with diselenide **228a** generating one equivalent of PhSe^+^ (**233**) and PhSe^•^ (**234**). Addition of the two selenium species **233/234** across the alkene followed by oxidation produces the benzylic carbocation **236** which undergoes reaction with the nucleophilic amine to yield the final product.

## Conclusion

This review summarized Fe-catalyzed domino coupling reactions of π-systems and illustrated the major breakthroughs in the field. Considerable developments have been accomplished in the field of iron catalysis providing non-toxic, inexpensive, and overall greener alternatives to precious metal catalysis. Despite the prevalence of precious metals in the field of catalysis, iron has proven a competitive rival for its reactivity in a variety of coupling reactions. Among them, significant progress has been made in cross-coupling reactions, proving competent with various electrophiles and organometallic reagents. Likewise, cross-dehydrogenative-coupling has offered a sustainable variant to traditional coupling reactions, with highly selective and fruitful coupling reactions being developed. Oxidative coupling and functionalization reactions have widely been used in rapidly increase molecular complexity for the addition of many different carbon and heteroatom functionalities.

Further work in this field will undoubtedly continue to expand the scope of potential coupling partners within Fe-catalyzed domino reactions. As with the majority of domino methodology, reactions are generally extremely substrate-dependent and rarely lead to the discovery of novel broad-scope reactivity; however, connecting known reactivity paradigms and reagents to substrates with varying propagation sites will lead to the development of novel complex scaffolds. Adoption of this methodology in total synthesis will further demonstrate the utility of these protocols and continue to advance the field’s scope and applicability. To date, a great deal of Fe-catalyzed multifunctionalization reactions employ styrene derivatives, or other activated π-systems, placing limitations on its relevance. Expanding the scope to include less reactive alkenes is necessary to see further advancements in this field. Although great progress has been made in the development of asymmetric variants, there is still significant room for improvement. The use of novel chiral ligands, or the use of chiral auxiliaries, could improve the scope of enantioselective cross-coupling and cross-dehydrogenative-coupling reactions.

While selectivity within multicomponent domino reactions is a challenging task, recent achievements have provided straightforward protocols for the construction and complex molecules through the generation of multiple carbon–carbon and carbon–heteroatom bonds in a single step. Increasing the selectivity and expanding the applicable substrate scope of Fe-catalyzed domino reactions is imminent. Future advancements of such methodology will make Fe-catalyzed domino reactions a mainstay in the organic chemist’s toolbox.
